# WhiA transcription factor provides feedback loop between translation and energy production in a genome-reduced bacterium

**DOI:** 10.3389/fmicb.2024.1504418

**Published:** 2024-12-23

**Authors:** Gleb Y. Fisunov, Vladimir B. Tsvetkov, Ekaterina A. Tsoy, Daria V. Evsyutina, Alexey D. Vedyaykin, Irina A. Garanina, Tatiana A. Semashko, Valentin A. Manuvera, Anna M. Varizhuk, Sergey I. Kovalchuk, Alexander I. Zubov, Nicolay A. Barinov, Olga V. Pobeguts, Vadim M. Govorun

**Affiliations:** ^1^Scientific Research Institute of Systems Biology and Medicine, Moscow, Russia; ^2^Federal Research and Clinical Center of Physical-Chemical Medicine of Federal Medical Biological Agency, Moscow, Russia; ^3^WCRC “Digital Biodesign and Personalized Healthcare”, Sechenov First Moscow State Medical University, Moscow, Russia; ^4^A.V. Topchiev Institute of Petrochemical Synthesis Russian Academy of Sciences, Moscow, Russia; ^5^Peter the Great St. Petersburg Polytechnic University, Saint Petersburg, Russia; ^6^Shemyakin-Ovchinnikov Institute of Bioorganic Chemistry, Russian Academy of Sciences, Moscow, Russia

**Keywords:** Mollicutes, mycoplasma, minimal cell, transcription factor, energy metabolism, ribosomal proteins

## Abstract

**Introduction:**

WhiA is a conserved protein found in numerous bacteria. It consists of an HTH DNA-binding domain linked with a homing endonuclease (HEN) domain. WhiA is one of the most conserved transcription factors in reduced bacteria of the class Mollicutes. Its function in Mollicutes is unknown, while it is well-characterized in *Streptomyces*. Here, we focused on WhiA protein from *Mycoplasma gallisepticum*.

**Methods:**

We used a combination molecular dynamics, EMSA, MST and AFM to study the DNA-binding and ATP-binding properties of WhiA from *M. gallisepticum*. The transcriptional repressor function of WhiA was demonstrated using gene knockdown, reporter constructs and proteome analysis.

**Results:**

We demonstrate that WhiA homolog from M. gallisepticum binds a conserved sequence of the GAYACRCY core (Y = C or T, R = A or G), which is located in the promoter of an operon coding for ribosomal proteins and adenylate kinase (*rpsJ* operon). We show that WhiA in *M. gallisepticum* is a repressor of *rpsJ* operon and a sensor of ATP. HTH domain binds to the core motif and HEN domain binds to the auxiliary motif GTTGT that is located downstream to the core motif. We show that binding by both domains to DNA is required to fulfill the transcription repressor function. Knockdown of whiA does not affect actively growing *M. gallisepticum*, but leads to the growth retardation after freezing.

**Discussion:**

We propose the following model for *M. gallisepticum* WhiA function. WhiA remains bound to the core motif at any conditions. At low ATP concentrations (starvation) HEN domain binds auxiliary motif and represses *rpsJ* operon transcription. At high ATP concentrations (nutrient-rich conditions) HEN domain binds ATP and releases auxiliary motif. It leads to the de-repression of *rpsJ* operon and increased production of ribosomal proteins.

## 1 Introduction

The regulation of housekeeping gene expression in simple organisms is of particular interest in synthetic biology. Bacteria with significantly reduced genomes like mycoplasmas (class Mollicutes) represent a good model of minimal cell. The study of gene expression control mechanisms in these organisms may help to understand the key principles of minimal but essential regulatory systems organization. Here, we focused on the WhiA transcription factor (TF) of *Mycoplasma gallisepticum*. The history of WhiA research dates back more than 50 years. Initially it was discovered in *Streptomyces coelicolor* among loci which mutations give rise to white colony phenotype. This phenotype was designated as *whi* (Hopwood et al., 1970). Mutations in *whiA* gene led to the incapability of sporulation due to the absence of sporulation septa in aerial hyphae, which was the cause of the respective phenotype (Chater, 1972). These regulators show remarkable conservation within bacteria, particularly in the gram-positive lineage of bacteria (Ainsa et al., 2000). They are found in a broad range of diverged clades, including Actinobacteria, Firmicutes, Mollicutes, Chlamydiae, and Bacteroidetes. These proteins consist of a helix-turn-helix (HTH) domain, which is common for bacterial TFs and a LAGLIDADG homing endonuclease (HEN) domain, which lacks nuclease activity (Kaiser and Stoddard, 2011). WhiA functions have been extensively studied in *Streptomyces*. It has been demonstrated that WhiA in *Streptomyces* plays a key role in sporulation regulation as a component of the complex regulatory network (Kaiser and Stoddard, 2011). This network includes TFs BldD, WhiA, WhiH, WhiI, and an alternative sigma factor, WhiG. The interplay between them determines if sporulation should be started or if vegetative growth should be continued (Kaiser and Stoddard, 2011; Xie et al., 2007). The binding site of WhiA has been identified in *Streptomyces* bacteria in different studies. Oligonucleotide binding competition analysis (Kaiser and Stoddard, 2011) and ChIP-seq (Bush et al., 2013) demonstrated that the WhiA binding site is a GACAC pentamer. DNA footprint analysis (Kaiser and Stoddard, 2011) showed that the protein binding area is much larger, consists of two protected regions, and is approximately 20 bp in length. It has been demonstrated that in *Streptomyces,* WhiA directly regulates multiple genes involved in cell division, including *parA*, *parB*, and *ftsZ*. In addition, it has been demonstrated that WhiA plays a role in chromosome segregation and integrity maintenance in *Bacillus subtilis* (Bohorquez et al., 2018), but shows non-specific DNA-binding activity and is not involved in sporulation (Surdova et al., 2013). WhiA knockout results in a viable phenotype in both *Streptomyces* (Bush et al., 2013) and *Bacillus* (Bohorquez et al., 2018). While *whiA* is not an essential gene in wild-type *B. subtilis*, it produces a lethal phenotype in a double knockout with the *parAB* operon (Bohorquez et al., 2018). Inactivation of *whiA* in *B. subtilis* affects the chromosome segregation (Bohorquez et al., 2023). In the mutant strain the distance between the nucleoids substantially increases. WhiA inactivation in *B. subtilis* affects transcriptional landscape as well (Bohorquez et al., 2023). However promoters directly regulated by WhiA were not identified. *Corynebacterium glutamicum* WhiA has been proven to bind DNA in a site-specific manner in complex with WhcD protein and regulates the expression of cell division genes (Lee et al., 2018).

WhiA is one of the four most universally conserved TFs in Mollicutes, together with HrcA, MraZ, and YebC/PmpR (Fisunov et al., 2016b). Its function is unknown, whereas high conservation indicates its importance. The role of WhiA in *Mycoplasma pneumoniae* was first proposed by Eilers in his PhD thesis (Eilers, 2010). He found that disruption of WhiA by transposon mutagenesis results in constitutive upregulation of *rpsJ* operon transcription, thus concluding that WhiA can be a regulator of the *rpsJ* operon. Eilers also found that the *rpsJ* operon of a WhiA knockout strain, in contrast to the wild-type strain, does not undergo repression at the stationary phase, indicating that WhiA serves as a sensor of the growth phase. Furthermore, the *rpsJ* promoter was identified as a target of WhiA in a ChIP-Seq study of *M. pneumoniae* (Yus et al., 2019). In the minimal synthetic mycoplasma genome (*Mycoplasma mycoides* JCVI-syn 3.0) *whiA* gene was retained (Hutchison et al., 2016). At least it indicates WhiA importance in the context of mycoplasma cell.

In this work, we studied the functions of WhiA with emphasis on its role in the minimal genome using the class Mollicutes bacterium *Mycoplasma gallisepticum*. Generally, Mollicutes feature a significant reduction in gene expression control mechanisms. On average, there are no more than 10 known regulators in mycoplasmas and other representatives of the most reduced clades of Mollicutes (Fisunov et al., 2016b). The more or less universally conserved core of TFs in Mollicutes includes regulators of cell division MraZ (Fisunov et al., 2016a), heat shock repressor HrcA (Chang et al., 2008), YebC/PmpR family TF with yet unknown function, and WhiA. The functional repertoire of regulators preserved during degenerative evolution is diverse in terms of function. MraZ is involved in the control of cytokinesis (Fisunov et al., 2016a), while HrcA mediates unfolded protein stress (Chang et al., 2008). Some of them, like HrcA, seem to be not essential for living in artificial conditions. WhiA, studied in this work, controls the core function of the cell, providing a feedback loop between energy metabolism and the synthesis of ribosome constituents.

## 2 Materials and methods

### 2.1 Cultivation, growth assay, genetic transformation and gene suppression of *Mycoplasma gallisepticum*

Briefly, *M. gallisepticum* strain S6 was grown in liquid medium (20 g/L tryptose, 3 g/L Tris, 5 g/L NaCl, 1.3 g/L KCl, 5% yeast dialysate, 10% horse serum, and 1% glucose at pH 7.4) at 37°C under aerobic conditions until the mid-exponential phase, as described in Mazin et al. (2014). All vectors used for transformation of *M. gallisepticum* were based on the pRM5L2 transposon vector used in a previous study (Matyushkina et al., 2016) and are listed in Supplementary Table S1. Transformation of *M. gallisepticum* was performed as described previously (Mazin et al., 2014).

Growth kinetics of *M. gallisepticum* was assayed using Varioskan Flash instrument (Thermo Fisher Scientific). Cells were grown in 96-well plates sealed with parafilm in 200 μL aliquots of growth medium at 37°C directly in the instrument. Each strain was grown in three technical replicates. Cell density was monitored at OD_640_ for 48 h.

For genetic transformation, RT-qPCR and proteome analysis *M. gallisepticum* was grown for two passages continuously and the cells in the exponential phase of the second passage were collected. 10% of the grown cultures were transferred to the second passage. Exponential phase corresponds to the 16 h of growth of wild-type *M. gallisepticum*. The growth phase was additionally monitored using Phenol Red indicator in the growth medium.

For genetic transformation, the cells were harvested by centrifugation at 8,000 g at 4°C for 10 min. The pellet was resuspended in 250 μL of electroporation buffer (8 mM HEPES, 272 mM sucrose, pH 7.4). The procedure was repeated twice to remove any traces of growth medium. Then, the cell suspension was transferred to an electroporation cuvette and the pulse was performed using a Gene Pulser device (Bio-Rad). The parameters for the cuvette and the pulse are as follows: 2 mm cuvette, voltage 2,500 V, resistance 100 *Ω*, and capacitance 25 μF. The cells were then transferred to 1 mL of fresh medium and grown for 4 h. Then, the cells were cultivated in a semi-liquid medium (identical to liquid growth medium) containing 0.3% agar and 2 μg/mL tetracycline until visible colonies were formed. The colonies were picked and grown in a liquid medium supplemented with 2 μg/mL tetracycline.

For artificial gene suppression, we used the dCas9 protein, where both endonuclease domains of wild-type *Streptococcus pyogenes* Cas9 were inactivated by mutation (Evsyutina et al., 2022). The binding of the dCas9:sgRNA complex to genomic DNA can block either transcription initiation or elongation depending on the location of the target sequence. The genes coding for dCas9 and sgRNAs were artificially synthesized and inserted into the pRM5L2 transposon vector. One vector carried one sgRNA gene. The subsequent transformation of *M. gallisepticum* was performed as described above. Vector content and integrity after transformation were assessed using Sanger sequencing. The strains transformed with the dCas9 vector without the sgRNA gene were used as a control.

### 2.2 Super-resolution microscopy

WhiA protein (Mgal-WhiA) was cloned from *M. gallisepticum* into transposon vector and fused with mMaple2 photoconvertible protein (Rumyantseva et al., 2019). The constructed vector was used to transform *M. gallisepticum* by electroporation as described above. The distribution of Mgal-WhiA within living *M. gallisepticum* cells were studied using SRRF method (Culley et al., 2018). An aliquot of 50 μL of *M. gallisepticum* cell culture in the late exponential growth phase was placed into a microscopy chamber. The chamber consisted of a coverslip, a slide and two stripes of double-side tape between them in order to form a channel in which solutions may be washed in and out. To facilitate better cell adhesion to the coverslip, the chamber was filled with poly-L-lysine solution for 10 min. Then the chamber was filled with cell suspension and was incubated for 1 h at 37°C upside down to let cells settle down to the coverslip. *M. gallisepticum* cells expressed the fusion of Mga-WhiA with mMaple2 protein (on C-terminus of WhiA) from the integrative vector.

Images were acquired using a cooled EM-CCD camera (Photometrics Cascade II) and oil-immersion objective lens with 100× magnification and the numerical aperture of 1.46 (Carl Zeiss). One pixel on raw images corresponded to 133 nm in the lens focal plane. Following filter sets were used: Semrock mCherry-40LP (for mMaple2 fluorescent protein excitation), Semrock DAPI (for mMaple2 switching from green to red state) and Semrock LF635/LP-B-000 [for fluorescence excitation of 5-SiR DNA specific dye (Bucevičius et al., 2019)]. For the fluorescence excitation a mercury lamp was used (X-cite 120Q). Ten images in the transmitted light channel were acquired, then 100 images in the channel of 5-SiR fluorescence, and then mMaple2 protein was converted into red by exposing the sample to DAPI fluorescence excitation light for 5 s. After that 100 images in the red channel of mMaple2 fluorescence were taken.

Image analysis was performed using ImageJ (Collins, 2007) or Fiji (Schindelin et al., 2012) software. Image series in transmitted light were averaged. Image series obtained in fluorescence channels of stained DNA and WhiA protein were processed by NanoJ SRRF plugin for ImageJ (Culley et al., 2018) to improve the spatial resolution obtained beyond the diffraction limit.

### 2.3 Recombinant protein purification

Recombinant WhiA from *M. gallisepticum* (Mgal-WhiA) was obtained as previously described (Fisunov et al., 2016a). The *whiA* gene was amplified from *M. gallisepticum* S6 genomic DNA and cloned into pET15b plasmid with an N-terminal His-tag and thrombin cut site using BamHI and SalI sites (cloning primers are listed in Supplementary Table S2). This resulted in the following amino acid sequence: MGSSH_6_SSGLVPRGS-[WhiA] (Supplementary Table S1). Amino acid substitutions were introduced into Mgal-WhiA through PCR mutagenesis. The substitutions were made for glycine in all the cases. All genetic engineering was carried out in the Top-10 strains of *Escherichia coli*. The proteins were overexpressed in *Escherichia coli* BL21-Gold (DE3) cells. Cells from an overnight culture were harvested by centrifugation, washed in PBS, and lysed by sonication using a Branson 250 Sonifier (Branson) at 22 kHz for 10 min. Proteins were obtained in a water-soluble form. The lysate was diluted with sample buffer containing 20 mM Na_2_HPO_4_, 10 mM imidazole, and 500 mM NaCl, pH 7.5. The protein was purified on a Tricorn 5/50 column (GE Healthcare) with Ni Sepharose High Performance (GE Healthcare) resin using the AKTA FPLC system (GE Healthcare). After the application of lysate, the column was washed with 25-mL aliquots of sample buffer, washed with wash buffer (20 mM Na_2_HPO_4_, 25 mM imidazole, 500 mM NaCl, pH 7.5), and finally with elution buffer (20 mM Na_2_HPO_4_, 500 mM imidazole, 500 mM NaCl, pH 7.5). The proteins were stored at −20°C in 50% glycerol.

### 2.4 Electrophoretic mobility shift assay

An aliquot of purified protein was incubated with 2.5 pmol of the FAM-or HEX-labeled ds-oligonucleotides for 10 min at 37°C. The full list of oligonucleotides used for EMSA is provided in Supplementary Table S2. The oligonucleotides were purchased from Lytech (Russia). The binding reaction for Mgal-WhiA was performed in 20 mM carbonate buffer (pH 9.8) with 6% glycerol. Electrophoresis was performed using a PROTEAN II xi electrophoretic cell (Bio-Rad) and a 6% acrylamide gel for 1 h at 450 V at 10°C. Mgal-WhiA was assayed in a carbonate buffer (pH 9.8). Gels were visualized using a ChemiDoc MP image system (Bio-Rad). The fluorescence was quantitated using Image Lab 5.1 software (Bio-Rad). The Kd values were calculated using the fractional saturation data (protein-shifted band fluorescence to the total fluorescence ratio) and the Hill model (Fraction bound = 1/(1 + (Kd/[WhiA])*^n^*)), where *n* is the Hill coefficient. EMSA images were analyzed using Fiji software (Schindelin et al., 2012).

### 2.5 Microscale thermophoresis assay

Same oligonucleotides as the ones used for EMSA were used for MST. For the analysis of WhiA-DNA interactions, pre-annealed stock solutions of the HEX-labeled ds-oligonucleotides in PBS buffer (10 mM sodium phosphate, pH 7.4, 140 mM NaCl, and 3 mM KCl) were mixed with protein solutions in PBS buffer supplemented with Tween-20 and imidazole to a final duplex concentration of 50 nM and protein concentration of 0–5 μM. For the analysis of WhiA-ATP interactions, the protein was labeled with Cy5 NHS-ester. Tween and imidazole concentrations in the final working buffer were 0.05% and 50 mM, respectively. The mixtures were incubated at 20°C for 15 min prior to MST measurements. MST curves were registered using a Monolith NT.115 (NanoTemper, Germany) equipped with a RED/GREEN detector with MST monitoring by HEX fluorescence in the GREEN mode. The dependence of the normalized HEX fluorescence after thermodiffusion on the protein concentration was analyzed using MO. Affinity analysis software (NanoTemper), and the microscopic dissociation constant (Kd) value was obtained by fitting the experimental data to the Hill model (Fraction bound = 1/(1 + (Kd/[WhiA])*^n^*)), where *n* is the Hill coefficient.

### 2.6 Forster’s resonance energy transfer assay

The pre-annealed BHQ1-labeled (Black Hole Quencher) duplex WhiA-Q3-F/WhiA-Q3_RC was mixed with the 6-FAM-labeled WhiA-F1/WhiA-F1_RC or WhiA-F2/WhiA-F2_RC (negative control) duplex in a 10 mM sodium phosphate buffer (pH 7.4) containing 140 mM NaCl and 3 mM KCl. The final concentration of each duplex was 1 μM. The protein was added to a final concentration of 1.5 μM. Fluorescence emission spectra were recorded before and after protein addition at 20°C upon excitation at 495 nm using a Chirascan spectrometer (Applied Photophysics, UK). Juxtaposition of the 6-FAM-labeled and BHQ-labeled duplexes upon binding with the protein was assessed based on 6-FAM fluorescence quenching by BHQ1.

### 2.7 RT-qPCR

Aliquots of the *M. gallisepticum* cell culture were directly lysed in TRIzol LS reagent (Life Technologies) at a 1:3 ratio of culture medium:TRIzol LS (v/v). The lysates were extracted with chloroform. The aqueous phase was used to precipitate the RNA by the addition of an equal volume of isopropanol followed by centrifugation. The resulting RNA was treated with DNAse I (Thermo Scientific), and cDNA was synthesized from random hexamer primers using Maxima Reverse Transcriptase (Thermo Scientific). Real-time PCR was performed using iQ SYBR Green Supermix (Bio-Rad) and a CFX96^™^ Real-Time PCR Detection System (Bio-Rad) PCR machine. The primers used for RT-qPCR are listed in Supplementary Table S2. Ct values were calculated using Bio-Rad CFX Manager software. The relative RNA level for each sample was determined using the 2^−∆∆Ct^ method and normalized to the level of enolase transcript (*eno*, GCW_02860) in the respective sample. qRT-PCR experiments were performed on three replicates per each transformant. At least two transformants were obtained per genetic construct. Primers are listed in Supplementary Table S2.

### 2.8 Proteomic analysis

Sample preparation for proteomic analysis was performed as follows: the samples were lysed in a lysis buffer containing 1% sodium deoxycholate (Sigma) and 100 mM Tris–HCl (pH 8.5) with a protease inhibitor cocktail (GE HealthCare) through ultrasonication with a Branson 1510 sonicator at 4°C for 1 min. Protein concentration was estimated using the BCA assay (Sigma). Aliquots containing 300 μg of the protein material were diluted to 1 μg/μL with lysis buffer, and Tris (2-Carboxyethyl) phosphine hydrochloride (TCEP, Sigma) and chloroacetamide (CAA, Sigma) were added to final concentrations of 10 and 30 mM, respectively. Cys-reduction and alkylation were achieved by heating the sample for 10 min at 85°C. Trypsin (Promega) was added at a ratio of 1:100 w/w to the protein amount and incubated at 37°C overnight. Then, the second trypsin portion 1:100 w/w was added, and the sample was incubated for 4 h at 37°C. Proteolysis was stopped by the addition of 1% trifluoroacetic acid. The precipitated sodium deoxycholate was then removed using ethyl acetate (Masuda et al., 2008). The samples were purified using OASIS columns (Waters) and analyzed by liquid chromatography-mass spectrometry (LC–MS).

LC–MS analysis was carried out on an Ultimate 3000 RSLC nano HPLC system connected to a QExactive Plus mass spectrometer (Thermo Fisher Scientific). Samples were loaded to a home-made trap column 20 × 0.1 mm, packed with Inertsil ODS3 3 μm sorbent (GL Sciences), in the loading buffer (2% ACN, 98% H_2_O, 0.1% TFA) at 10 μL/min flow and separated at RT in a home-packed fused-silica column 500 × 0.1 mm packed with Reprosil PUR C18AQ 1.9 (Dr. Maisch) into the emitter prepared with P2000 Laser Puller (Sutter) (Kovalchuk et al., 2019). Samples were eluted with a linear gradient of 80% ACN, 19.9% H_2_O, 0.1% FA (buffer B) in 99.9% H_2_O, 0.1% FA (solvent A) from 4 to 36% of solvent B in 1 h at 0.44 μL/min flow at 20°C.

MS data were collected in DDA mode. MS1 parameters were as follows: 70 K resolution, 350–2,000 scan range, maximum injection time 50 ms, AGC target value 3 × 10^6^. Ions were isolated with 1.4 m/z window and 0.2 m/z offset targeting 10 highest intensity peaks of +2 to +6 charge, 8 × 10^3^ minimum AGC, preferred peptide match and isotope exclusion. Dynamic exclusion was set to 40 s. MS2 fragmentation was carried out in HCD mode at 17.5 K resolution with 27% NCE. Ions were accumulated for maximum 45 ms with target AGC 1 × 10^5^.

Identification of the DDA files was performed with the MaxQuant 1.6.6.0 software with default settings against the *M. gallisepticum* S6 Uniprot reference database with two additional protein sequences: TetM (tetracycline resistance protein encoded by the vector) and Cas9 of *S. pyogenes*. Fold change, Student’s t-test and Benjamimi-Hochberg correction were calculated using R version 3.6.1. The proteomic data is available via PRIDE database, project ID PXD026928.^1^

Differential abundance analyses of proteins in *whiA* knockdown and *whiA*-overexpressing transformants versus *dcas9*-expressing transformants of *M. gallisepticum* S6 were performed using the empirical Bayes method available in DEP (uses limma). The resulting *p*-values were adjusted using the Benjamini-Hochberg approach and the significance threshold was set at an adjusted *p*-value of 0.05 and a log_2_(fold change) of 0.5. Two transformants with *whiA* knockdown, *whiA*-overexpression and *dcas9*-expression were used. Proteomic experiments were performed on three replicates per each transformants.

### 2.9 Atomic force microscopy

For preparation of the GM-HOPG surface, 10 μL of 0.01 g/L [Gly_4_-NHCH_2_]_2_C_10_H_20_ (GM, Nanotuning, Russia) solution in water was deposited onto a freshly cleaved HOPG (ZYB quality, mosaic spread 0.8–1.2°, NanoAndMore, Switzerland, and NT-MDT, Russia) surface for 10 min, then supplemented with 100 μL of Milli-Q water and dried with a nitrogen flow.

The Mgal-WhiA mixture with DNA was prepared in the same manner as for the EMSA. The DNA fragment with the *rpsJ* promoter was amplified from the genomic DNA of *M. gallisepticum* (primers are listed in Supplementary Table S2). For the AFM study under ambient conditions, 0.5 μL sample solution was deposited onto the GM-HOPG surface for 1 s, followed by the addition of 100 μL of deionized water for 10 s. Subsequently, the droplets were removed by nitrogen flow.

AFM imaging under ambient conditions was performed using a multimode atomic force microscope, Ntegra Prima (NT-MDT), operated with ultrasharp tips (carbon nanowhiskers with a curvature radius of several nanometers grown at tips of commercially available silicon cantilevers with a spring constant of 5–30 N/m) in an attraction regime of intermittent contact mode (Klinov and Magonov, 2004). The line scan rate was typically 1 Hz with 1,024 × 1,024 pixels per image. The images were analyzed using Nova software (NT-MDT).

### 2.10 Luciferase assay

The standard mixtures for EMSA containing 1 mM ATP were prepared with or without the addition of the Mgal-WhiA protein. The samples were then incubated for 60 min at 37°C. ATP concentration was measured using a luciferase assay. The measurements were carried out on a Lum 1200 chemiluminometer (DiSoft, Russia). One microliter of the sample was transferred to 200 μL of buffer containing 50 mM potassium phosphate buffer, 5 mM MgCl_2_, and 5 μL of luciferin-luciferase mixture (Lumtek, Russia) at pH 7.8. Measurements were performed in triplicate.

### 2.11 Docking and molecular dynamics

The 3D models of Mgal-WhiA, *rpsJ* promoter DNA, and ATP were built using the molecular graphics software package in Sybyl-X software (Certara). The Mgal-WhiA 3D model was built by homology using the resolved 3D structure of WhiA from *Thermotoga maritima* 3hyi.1. The same fragment of the *rpsJ* promoter used for the EMSA was used for modeling. Partial charges on the ATP atoms were calculated according to the following scheme: First, in order to find the most minimal conformation, scanning of the conformational space of the ATP was performed with the application of the molecular mechanical approach and Monte Carlo method using Molsoft ICM-Pro 3.8.6 (Abagyan et al., 1994). To calculate the interatomic interactions, a force field mmff (Halgren, 1996) was used at this stage. Further optimization of the conformation found at the first step, for the purpose of searching geometry with the smallest energy, and calculation of electron density distribution were performed using a second-order *Møller*–*Plesset* perturbation theory (MP2) (Møller and Plesset, 1934) and sdd basis sets. Then, the Merz-Singh-Kollman scheme (Singh and Kollman, 1984) was applied to obtain the electron density distribution for calculation of the grid for the electrostatic potential fitting with the following parameters: (6/41 = 10), the number of surfaces around the atoms, and (6/42 = 17)—the density of test points on these surfaces. The restrained electrostatic potential (RESP) method (Bayly et al., 1993) was applied to fit the grid obtained in the previous step to calculate the partial atomic charges. All quantum mechanics simulations were performed using the Gaussian 09 program (Frisch et al., 2009).

To define the most probable binding site of ATP on the WhiA surface, flexible ligand docking was performed using ICM-Pro 3.8.6. Docking was performed in two stages. During the first procedure, the geometry of WhiA did not change. Before starting a docking procedure the structures of WhiA and ATP were converted into an ICM object. According to the ICM method, the molecular models were described using internal coordinates as variables. The parameters needed for the interatomic energy calculation and the partial charges for the atoms of WhiA were taken from ECEPP/3 (Arnautova et al., 2006). The biased probability Monte Carlo (BPMC) minimization procedure (Abagyan and Totrov, 1994) was used for global energy optimization. From the random conformations formed during “rigid” docking, 100 with the best binding energy scoring were selected. When the second docking procedure was carried out, the confirmations received during the first procedure were used as starting materials. The target-ligand complexes obtained from the first rigid-body docking were further refined by optimizing the conformation of side-chain amino acids located in the vicinity of the 4 Å radius of the ligand using the BPMC procedure.

The most probable conformation of complex WhiA-DNA was also carried out in two stages using a docking procedure. In the first stage, “rigid” docking with unchanged conformations of WhiA and DNA was used with the application of Hex 8.0.0 (Macindoe et al., 2010). The search for the most probable location of DNA on the target WhiA was carried out by analyzing the steric and electrostatic correlation between them in conjunction with post-processing MM minimization using the OPLS force field. The parameters for the docking procedure were selected as follows: FFT Mode—3D, receptor range—180, ligand range—180, twist range—360, distance range—40, angular increments—7.5 for the ligand and the target rotational angles, and 5.5 for twist angles. In the second stage, the 100 complexes, obtained in the first stage and selected by the scoring function during post-processing, were minimized using SYBYL X (Certara) and the Powell method (Powell, 1977). The following settings were used: parameters for interatomic interactions and partial charges on the atoms from the Amber 7ff02 force field, a non-bonded cut-off distance of 8 Å, a distance-dependent dielectric function, the number of iterations equal to 500, the simplex method in an initial optimization, and an energy gradient convergence criterion of 0.05.

MD simulations were performed using a suite of programs in Amber18 (Case et al., 2018). Influence of the solvent was simulated with the application model of water molecules OPC3 (Izadi and Onufriev, 2016). The simulation was performed using periodic boundary conditions and a rectangular box. The buffer between the WhiA-DNA and WhiA-ATP complexes and the periodic box wall was at least 15 Å. The parameters needed for the interatomic energy calculation were taken from the force fields OL15 (Krepl et al., 2012; Zgarbová et al., 2013; Zgarbová et al., 2015) for DNA, ff14SBonlysc force field (Maier et al., 2015) for the protein, and gaff2 for ATP. K+ ions were used to neutralize the negative charge. Аt the beginning of computing the investigated systems were minimized by two steps. In the first stage, the location of the solvent molecules was optimized using 1,000 steps (500 steps of steepest descent followed by 500 steps of conjugate gradient), at which the mobility of all solute atoms was restrained with a force constant of 500 kcal × mol^−1^ × Å^−2^. In the second stage, the optimization was performed without any restriction using 2,500 steps (1,000 steps of steepest descent, 1,500 steps of conjugate gradient). Then, gradual heating to 300 K was performed for 20 ps. To avoid wild fluctuations for the investigated systems in this stage, weak harmonic restraints were used with a force constant of 10 kcal × mol^−1^ × Å^−2^ for all atoms that were not a part of the solvent. The SHAKE (Ryckaert et al., 1977) algorithm was applied to constrain bonds to hydrogen atoms, which allowed the use of a 2 fs step. Scaling of non-bonded 1–4 van der Waals and electrostatic interactions was performed using standard Amber values. The cutoff distance for non-bonded interactions was 10 Å, and the long-range electrostatics were calculated using the particle mesh Ewald method (Darden et al., 1993). The MD simulations in the production phase were carried out using constant temperature (T = 300 K) and constant pressure (*p* = 1 atm) over 80 ns. To control the temperature, a Langevin thermostat was used with a collision frequency of 1 ps^−1^. The energies of the complex WhiA-DNA were estimated using the GBSA approach. The polar contribution (EGB) was computed using the generalized Born (GB) method and the algorithm developed by Onufriev et al. to calculate the effective Born radii (Onufriev et al., 2000). The nonpolar contribution to the solvation energy (Esurf), which includes solute-solvent van der Waals interactions and the free energy of cavity formation in the solvent, was estimated from the solvent-accessible surface area (SASA). To simulate the ensemble of ATP, the interaction with WhiA, the distribution of ATP in the rectangular box was modeled using the PACKMOL package (Martínez et al., 2009). When analyzing the vicinity, the distance between an amino acid and a given DNA region at a given trajectory step was determined by the following algorithm: the distances between the atoms of the amino acid under consideration and the nucleotide atoms included in this DNA region, including hydrogens, were calculated in pairs, and the minimum was found from this array. Contact was considered valid if the calculated distance did not exceed 3 Å. Similarly, the vicinity between the atoms of the protein and ATP was determined.

## 3 Results

### 3.1 *Mycoplasma gallisepticum* WhiA protein co-localizes with the nucleoid

We studied the distribution of WhiA protein (Mgal-WhiA) and genomic DNA (the nucleoid) in *M. gallisepticum* cells using super-resolution microscopy (SRRF) within the living cells. The WhiA protein labeled by phusion with mMaple2 protein was expressed from the integrative vector. On the obtained images WhiA and genomic DNA formed compact foci that co-localized with each other (Supplementary Figures S1, S2). Dividing cells formed two foci, respectively. Thus we conclude that WhiA of *M. gallisepticum* features non-specific DNA-binding activity similar to the one described for *B. subtilis* WhiA (Surdova et al., 2013).

### 3.2 The rpsJ operon of multiple bacteria features a conserved motif in its promoter

Multiple phylogenetic groups of bacteria (Firmicutes, Mollicutes, Actinobacteria, Thermotogae, Bacteroidetes, and Chlamydiae) feature a conserved operon of ribosomal protein genes, the first of which is *rpsJ* (S10 ribosomal protein) in the majority of species. Here in, we will refer to it as the *rpsJ* operon. It encodes ribosomal proteins and several other proteins. The exact genetic content of the operon depends on the species, but it may contain over 30 genes (Supplementary Figure S3A). Analysis of the intergenic region upstream to the *rpsJ* operon of the bacteria that retained the *whiA* gene revealed a conserved sequence with the GAYACRCY core (Y = C or T, R = A or G) in the majority of species (Supplementary Figure S3B; Supplementary Table S3). At the same time, there are clade-specific preferences for redundant nucleotides. More GC-rich genomes tend to have an increased GC-composition of the motif, and vice versa. The sequence is homologous to the WhiA binding site found in *Streptomyces* in the promoters of genes involved in sporulation and the *whiA* gene itself (Kaiser and Stoddard, 2011), (Bush et al., 2013). Some clades of bacteria feature an additional clade-specific conserved motif downstream of the core (Supplementary Figure S3B). In Firmicutes and Mollicutes, this sequence is similar to the -35-box of the sigma-70 bacterial promoter. Here in, we refer to it as an auxiliary (aux) motif.

### 3.3 WhiA is a transcriptional repressor of rpsJ operon and plays role in transition from growth stop to active growth in *Mycoplasma gallisepticum*

**Figure 1:**
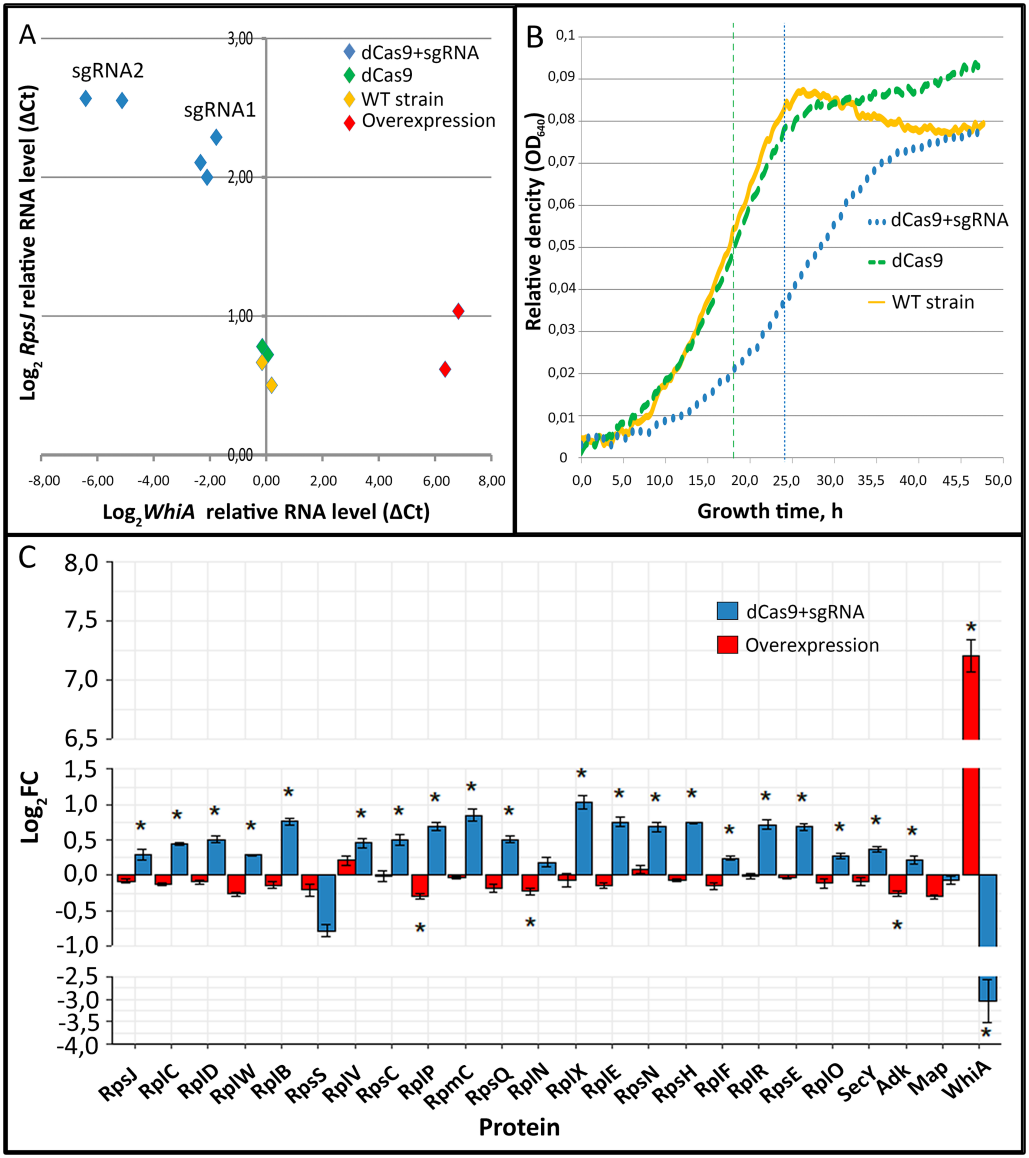
The effects of *whiA* knockdown and overexpression on *rpsJ* operon transcription **(A)** and translation **(C)** in *Mycoplasma gallisepticum.* **(A)** The effects of *whiA* knockdown and overexpression on *rpsJ* operon mRNA level in *M. gallisepticum*. The transcription was measured by RT-qPCR. RNA level was calculated relatively to enolase (*eno*) transcript. The respective primers are listed in Supplementary Table S2. Each dot corresponds to the independently obtained transformant strain. The repression was carried out using CRISPR interference. Two sgRNAs with different sequences against *whiA* were used (sgRNA1 and sgRNA2). For the detailed information on the vectors see Supplementary Table S2. The strains with dCas9 overexpression were used as a control as well as two biological replicates of the wild-type strain. **(B)** Growth curves (OD_640_) of WT *M. gallisepticum*, *whiA* knockdown strains and dCas9 control strains in the growth reactivation experiment. **(С)** The effect of *whiA* repression and overexpression on *rpsJ* operon-encoded proteins abundance in *M. gallisepticum*. Protein abundance was measured using LC–MS proteomics and the LFQ method. The same strains as shown on the panel **(A)** were used for the proteome analysis. For the proteome analysis only sgRNA2 expressing strains (the most effective knockdown) were used. dCas9 expressing strains without sgRNA gene were used as a control. Asterisks (*) indicate differentially expressed proteins with a *p*-value < 0.05. Standard error is indicated by error bars. Proteins are presented in the same order as encoded in the *rpsJ* operon. The change of the WhiA protein abundance is shown for comparison. The complete quantitative data on the proteomes of the control strains (dCas9 only), knockdown strains (dCas9 + sgRNA) and overexpression strains are provided in the Supplementary Table S4.

To elucidate WhiA function we performed *whiA* gene knockdown in *M. gallisepticum* by dCas9 protein via CRISPR interference (Evsyutina et al., 2022) and overexpression using a previously designed vector system (Matyushkina et al., 2016). Gene knockdown via dCas9 did not lead to complete gene loss, but suppressed transcription to different extents depending on the sgRNA design and dCas9 expression. We designed a set of sgRNAs and obtained a set of *M. gallisepticum* transformants with different suppression of *whiA* gene transcription. The overexpression of *whiA* was 1.5–2 orders of magnitude. The suppression of *whiA* ranged from one to two orders of magnitude. We demonstrated that the suppression of *whiA* gene transcription was strongly correlated with the upregulation of *rpsJ* operon transcription with a Spearman coefficient of −0.89 (Figure 1A), while overexpression had no effect. For the further work we used sgRNA2 only as the most effective.

The obtained *whiA* knockdown strains of *M. gallisepticum* did not demonstrate phenotypic differences compared to dCas9 expressing strains when grown under standard conditions. In particular the growth rate was identical if the cells were continuously passaged. To elucidate the *whiA* knockdown effect we performed growth reactivation test. The cells were grown until the stationary phase to reach the maximum cell density. Then the cells were frozen for a week at −20°C to stop metabolic processes. Then the cells were thawed and passaged to a fresh medium and the growth rate was monitored (Figure 1B; Supplementary Table S4). After the freezing the growth rate of the dCas9 expressing strains was the same as of the wild-type *M. gallisepticum*. The *whiA* knockdown strains demonstrated substantial growth retardation. They reached the mid-log phase on average 6 h later and the stationary phase 10 h later than the dCas9 expressing strains (Figure 1B). We propose that WhiA is important during translation reactivation after the growth stop under unfavorable conditions. *M. gallisepticum* lacks spores or other specialized dormant forms. However it can survive for days on feathers and in the environment (Yavari et al., 1994). Thus it may need mechanisms to attenuate biosynthetic processes to the low temperature and the lack of nutrients.

Quantitative proteome analysis indicated that the knockdown of *whiA* leaded to the upregulation of *rpsJ* operon on the protein level as well (Figure 1C; Supplementary Table S5). Consequently it resulted in the distortion of ribosomal proteins stoichiometry in cell. The *rpsJ* operon upregulation in *M. gallisepticum* was observed in the exponential phase. In contrast in *M. pneumoniae* the upregulation of *rpsJ* operon after *whiA* transposon knockout was observed only in the stationary phase (Eilers, 2010). Thus in *M. pneumoniae* the *rpsJ* operon promoter is de-repressed in the exponential phase and repressed in the stationary phase, while in *M. gallisepticum* it remains repressed constitutively. The overexpression of WhiA did not affect *rpsJ* operon transcription, probably due to its saturation concentration in the wild-type strain. Thus, we conclude that the function of WhiA in *M. gallisepticum* is essentially the same as that in *M. pneumoniae*: the repression of *rpsJ* operon transcription. However, the intracellular conditions that regulate WhiA activity are different in these species.

### 3.4 WhiA specifically binds conserved sequence in the promoter of the rpsJ operon

**Figure 2:**
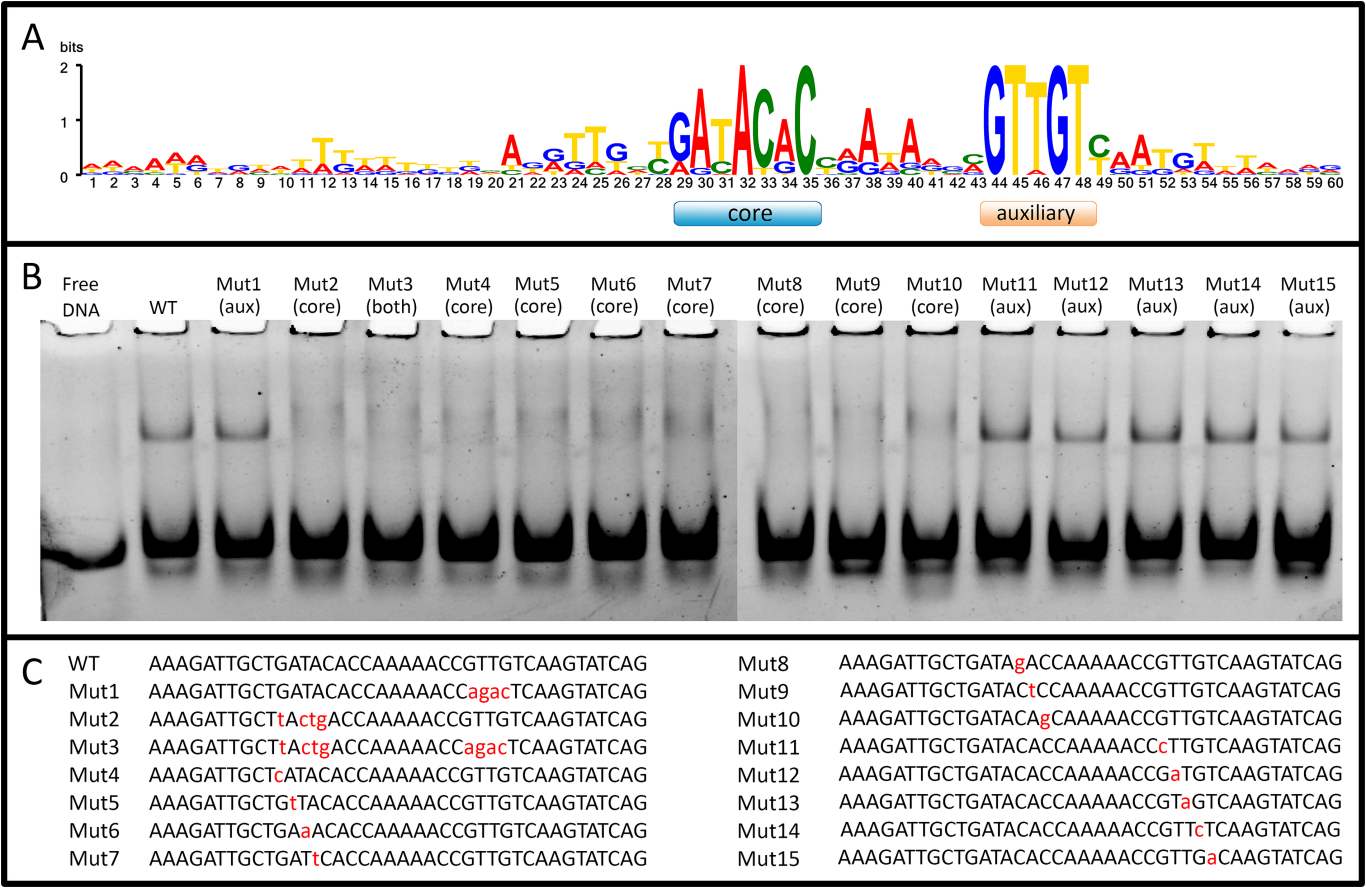
WhiA binding site in *M. gallisepticum*. **(A)** The sequence logo of WhiA binding sites in the *rpsJ* operon promoter of Mollicutes. **(B)** EMSA of Mgal-WhiA recognition of wild-type and mutated binding sites. 125 nM of oligonucleotide and 300 nM of Mgal-WhiA were used for the analysis. **(C)** Oligonucleotide sequences used for EMSA. The positions of the nucleotide substitutions (relatively to the consensus) are shown in lower case.

We obtained recombinant WhiA protein from *M. gallisepticum* (Mgal-WhiA) and used the EMSA and microscale thermophoresis (MST) assays to study their DNA binding properties. To confirm the binding specificity of Mgal-WhiA (Figure 2; Supplementary Figure S4) we used a set of 40-mer ds-oligonucleotides corresponding to their predicted binding sites with core and aux motif (Figure 2A) of the wild-type sequence (WT oligonucleotide), with mutations at the conserved positions (Figures 2B,C) or a foreign promoter sequence—negative control (Supplementary Figure S4). Mgal-WhiA showed noticeable binding activity with any DNA. The binding affinity and the complex mainly formed depend on the DNA sequence. Mgal-WhiA was able to form two complexes with 40-mer oligonucleotides: low molecular weight and high molecular weight. The ratio between the low-molecular and high-molecular complexes of Mgal-WhiA was strongly dependent on DNA sequence. A detailed study of Mgal-WhiA revealed that it was sensitive to single-nucleotide substitutions within the core motif. Disruption of the auxiliary -35-like motif imposed no observable effect on its ability to recognize the specific DNA if the core motif remained intact.

**Figure 3:**
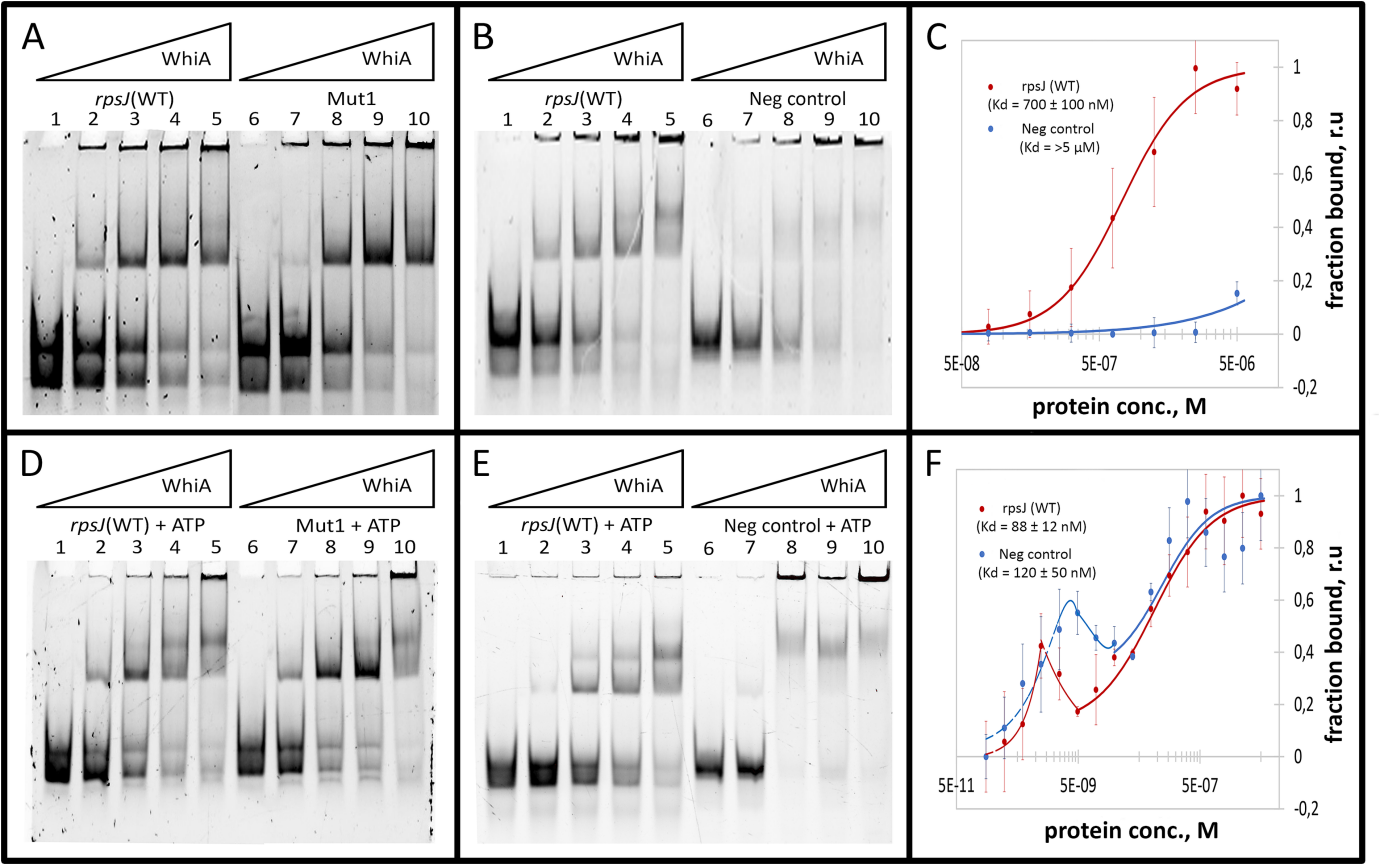
Mgal-WhiA K_d_ determination in the absence and in the presence of 1 mM ATP. **(A)** Titration of the oligonucleotide with wild-type Mgal-WhiA binding site from the *rpsJ* operon promoter (rpsJ WT) in parallel with the oligonucleotide with mutated aux motif (Mut1). The bands are schematically labeled on the left (from bottom to top): labeled ss-oligonucleotide, ds-oligonucleotide, the monomeric complex, the dimeric complex. Lanes: 1 – free rpsJ WT oligonucleotide 250 nM (same concentration for all lanes), 2 – 360 nM Mgal-WhiA, 3 – 720 nM Mgal-WhiA, 4 – 1,080 nM Mgal-WhiA, 5 – 1,435 nM Mgal-WhiA, 6 – free Mut1 oligonucleotide 250 nM, 7 – 360 nM Mgal-WhiA, 8 – 720 nM, 9 – 1,080 nM Mgal-WhiA, 10 – 1,435 nM Mgal-WhiA. **(B)** Titration of the oligonucleotide with wild-type Mgal-WhiA binding site from the *rpsJ* operon promoter (rpsJ WT) in parallel with the negative control oligonucleotide (Neg. control). Lanes: 1 – free rpsJ WT oligonucleotide 250 nM, 2 – 360 nM Mgal-WhiA, 3 – 720 nM, 4 – 1,080 nM Mgal-WhiA, 5 – 1,435 nM Mgal-WhiA, 6 – free negative control oligonucleotide 250 nM, 7 – 360 nM Mgal-WhiA, 8 – 720 nM, 9 – 1,080 nM Mgal-WhiA, 10 – 1,435 nM Mgal-WhiA. **(C)** Determination of Mgal-WhiA binding constant by microscale thermophoresis (MST) for wild-type binding site (*rpsJ* WT) and negative control (Neg. control) oligonucleotides. **(D)** Titration of the oligonucleotide with wild-type Mgal-WhiA binding site from the *rpsJ* operon promoter (rpsJ WT) in parallel with the oligonucleotide with mutated aux motif (Mut1) in the presence of 1 mM ATP. Lanes: 1 – free rpsJ WT oligonucleotide 250 nM (same concentration for all lanes), 2 – 360 nM Mgal-WhiA, 3 – 720 nM, 4 – 1,080 nM Mgal-WhiA, 5 – 1,435 nM Mgal-WhiA, 6 – free Mut1 oligonucleotide 250 nM, 7 – 360 nM Mgal-WhiA, 8 – 720 nM, 9 – 1,080 nM Mgal-WhiA, 10 – 1,435 nM Mgal-WhiA. **(E)** Titration of the oligonucleotide with wild-type Mgal-WhiA binding site from the *rpsJ* operon promoter (*rpsJ* WT) in parallel with the negative control oligonucleotide (Neg. control) in the presence of 1 mM ATP. Lanes: 1 – free rpsJ WT oligonucleotide 250 nM, 2 – 360 nM Mgal-WhiA, 3 – 720 nM, 4 – 1,080 nM Mgal-WhiA, 5 – 1,435 nM Mgal-WhiA, 6 – free negative control oligonucleotide 250 nM, 7 – 360 nM Mgal-WhiA, 8 – 720 nM, 9 – 1,080 nM Mgal-WhiA, 10 – 1,435 nM Mgal-WhiA. **(F)** Determination of Mgal-WhiA binding constant by microscale thermophoresis (MST) for the wild-type binding site (*rpsJ* WT) and negative control (Neg. control) in the presence of 1 mM ATP.

The binding constant (Kd) was measured for Mgal-WhiA with specific and non-specific ds-oligonucleotides by MST and was calculated from EMSA data (Figures 3A–C; Supplementary Figure S5). The MST data were obtained for the equilibrium of low- and high-molecular complexes because they could not be separated using this approach. The Kd measured by MST was 700 ± 100 nM for the WT sequence and > 5 μM for the negative control DNA. The binding curve of Mgal-WhiA to the specific sequence bears signs of positive cooperativity (*n* = 1.8 ± 0.2, where n is the Hill coefficient), which indicates a possible 2:1 or higher binding stoichiometry. Thus, we assumed that the high-molecular complex represents a dimer of Mgal-WhiA, while the low-molecular complex corresponds to a monomer. However, the dimer complex may represent two Mgal-WhiA proteins independently bound to the same oligonucleotide. To verify whether Mgal-WhiA oligomerization is DNA-dependent, we performed EMSA with the Cy5 labeled Mgal-WhiA protein and found no signs of the oligomerization without DNA. Thus, we proposed that the formation of the Mgal-WhiA dimer preferably occurs on DNA rather than in solution. We hypothesized that the oligomerization state of Mgal-WhiA on DNA is limited by DNA fragment length and is a result of its non-specific DNA-binding activity, e.g., the multiple copies of Mgal-WhiA can successively bind the same DNA molecule until there is free space on it. Thus the formation of monomeric or dimeric complex with 40-mer oligonucleotide depends on the space occupied by first bound Mgal-WhiA molecule. Since WhiA features two DNA-binding domains it can bind DNA in two conformations by one domain only or by both domains simultaneously. We proposed that the binding by both domains leaves no free space on the 40-mer oligonucleotide for the second molecule, which result in the formation of the monomeric low-molecular complex. If the first Mgal-WhiA molecule binds by only one of the domains the second Mgal-WhiA molecule can still bind the oligonucleotide and form the dimeric high-molecular complex.

Further we measured binding constant of WhiA to WT sequence and the sequence with mutated aux motif (Mut1) using EMSA. The fraction saturation data from the EMSA experiments allowed us to calculate the total binding constant using an orthogonal approach. The binding constants for the WT and Mut1 oligonucleotides was 700 ± 80 nM and 750 ± 30 nM, respectively. The binding constant for negative control DNA identified by EMSA was >5 μM. The data obtained by EMSA are in good agreement with the data obtained by MST, resulting in a difference of approximately an order of magnitude in the Kd for the specific and non-specific binding. Considering that the binding constants for the WT and the aux-mutated (Mut1) oligonucleotides are essentially the same, we conclude that the core motif contributes the most to the DNA-binding and motif recognition functions of Mgal-WhiA. Thus, we conclude that Mgal-WhiA preferably recognizes core motif GATACACC. This core motif is highly homologous with the WhiA binding site identified in *Streptomyces coelicolor* (Kaiser and Stoddard, 2011).

### 3.5 WhiA is a sensor of ATP

Previously, it was demonstrated that homing endonucleases depend on ATPase activity for DNA hydrolysis (Guhan and Muniyappa, 2003). The adenylate kinase (*adk*) gene (coding for an enzyme that interconverts ATP and AMP into ADP and vice versa) is a conserved component of the *rpsJ* operon (Supplementary Figure S3A). In *M. pneumoniae*, WhiA-dependent repression occurs in the stationary phase, which indicates that it is dependent on some intracellular marker of starvation. Taking these facts into account, we proposed that WhiA could have retained ATPase or ATP-binding function and therefore may be representative of the concentration of ATP in cells. The concentration of ATP in *E. coli* was measured and resided within a range of 1–5 mM (Yaginuma et al., 2014). We performed an EMSA study with different nucleoside mono-, di-, and triphosphates as ligands for Mgal-WhiA at a 1 mM concentration, using phosphate buffer as a control. In EMSA studies NTPs shifted the equilibrium between dimer and monomer toward the dimer in the most pronounced manner; the shift induced by NDPs was much less, but still detectable; NMPs and free phosphate did not induce any effect (Supplementary Figure S6). Among the NTPs, the strongest effect was induced by ATP. Therefore, we selected ATP as the preferred ligand. The obtained data indicate that Mgal-WhiA binds ATP at millimolar concentrations. The Kd of ATP binding to Mgal-WhiA measured by MST was 5 ± 0.1 mM and the Hill coefficient was 2.76 indicating possible cooperative binding (e.g., three ATP molecules per Mgal-WhiA, Supplementary Figure S7).

As described above, the deletion of the auxiliary motif results in an equilibrium shift toward the monomer. In the presence of ATP, this shift remained proportional, for example, ATP induced a shift toward the dimer with Mut1 oligonucleotide as well as with WT, but at higher concentrations of the protein (Figures 3D–F). We then measured the Kd of the complex in the presence of ATP using MST and calculated it from the EMSA data (Figures 3D–F; Supplementary Figure S5). The resulting MST curves suggest a multistep binding process. The interactions between Mgal-WhiA and WT oligonucleotide appear to be followed by a conformational rearrangement of the complex in the low nanomolar concentration range, as evidenced by the fluorescence intensity “spike.” Further binding interactions were complied with the Hill model (Kd = 88 ± 12 nM; *n* = 1). In the case of the negative control oligonucleotides, the rearrangement occurs at low to medium nanomolar concentrations, and subsequent binding is characterized by a Kd of 120 ± 50 nM. The Kd values obtained from the EMSA data were 680 ± 90 and 430 ± 90 for the WT and Mut1 oligonucleotides, respectively. For the negative control, it was still >5 μM. Thus, we conclude that ATP does not affect the overall binding constant, but induces conformational changes in Mgal-WhiA, which leads to a shift in equilibrium between the dimer and the monomer. Since the EMSA data allows a separate account of the monomer and dimer titration curves, we used them to calculate Kd values specifically for the monomers. For the WT oligonucleotide, the monomer Kd was 610 ± 100 nM in the absence of ATP and 240 ± 70 nM in the presence of ATP. For the Mut1 (mutated aux) oligonucleotide, the monomer Kd was 850 ± 130 nM and 290 ± 180 nM with and without ATP, respectively. Thus, ATP enhanced Mgal-WhiA binding to the core sequence, irrespectively of the aux motif. This process was accompanied by a more effective dimer formation (Supplementary Figures S9A,B). At the same time, the aux motif affected the equilibrium between the dimer and monomer by shifting it toward the dimer.

We measured the putative ATPase activity of Mgal-WhiA using luciferase assay (Supplementary Figure S10). We identified Mgal-WhiA lacked ATPase activity. Thus, we conclude that Mgal-WhiA binds ATP as a ligand, but does not hydrolyze it and does not use it as an energy source for the conformational change. The upregulation of Mgal-WhiA expression in *M. gallisepticum* via the artificial genetic construct did not result in any changes in *rpsJ* operon transcription (Figure 1A). Thus, we conclude that Mgal-WhiA activity in cells is predominantly regulated by ATP rather than by protein concentration.

*M. gallisepticum* demonstrated a transient increase in ATP concentration upon treatment with the proton ionophore carbonyl cyanide 3-chlorophenylhydrazone (CCCP). The increase in ATP level was measured using a luciferase assay (Supplementary Figure S11). While the mechanistic nature of this phenomenon is unclear, it can be used as an instrument to rapidly increase ATP level in cells. The change in the transcriptional landscape of *M. gallisepticum* during CCCP stress was measured previously by Semashko et al. (2017). CCCP treatment induced a statistically significant upregulation of the majority of *rpsJ* operon genes. The average upregulation was slightly more than two-fold, which is within the range of WhiA-mediated regulation according to the CRISPR interference data.

### 3.6 Atomic force microscopy supports specific and non-specific DNA-binding activity of Mgal-WhiA

**Figure 4:**
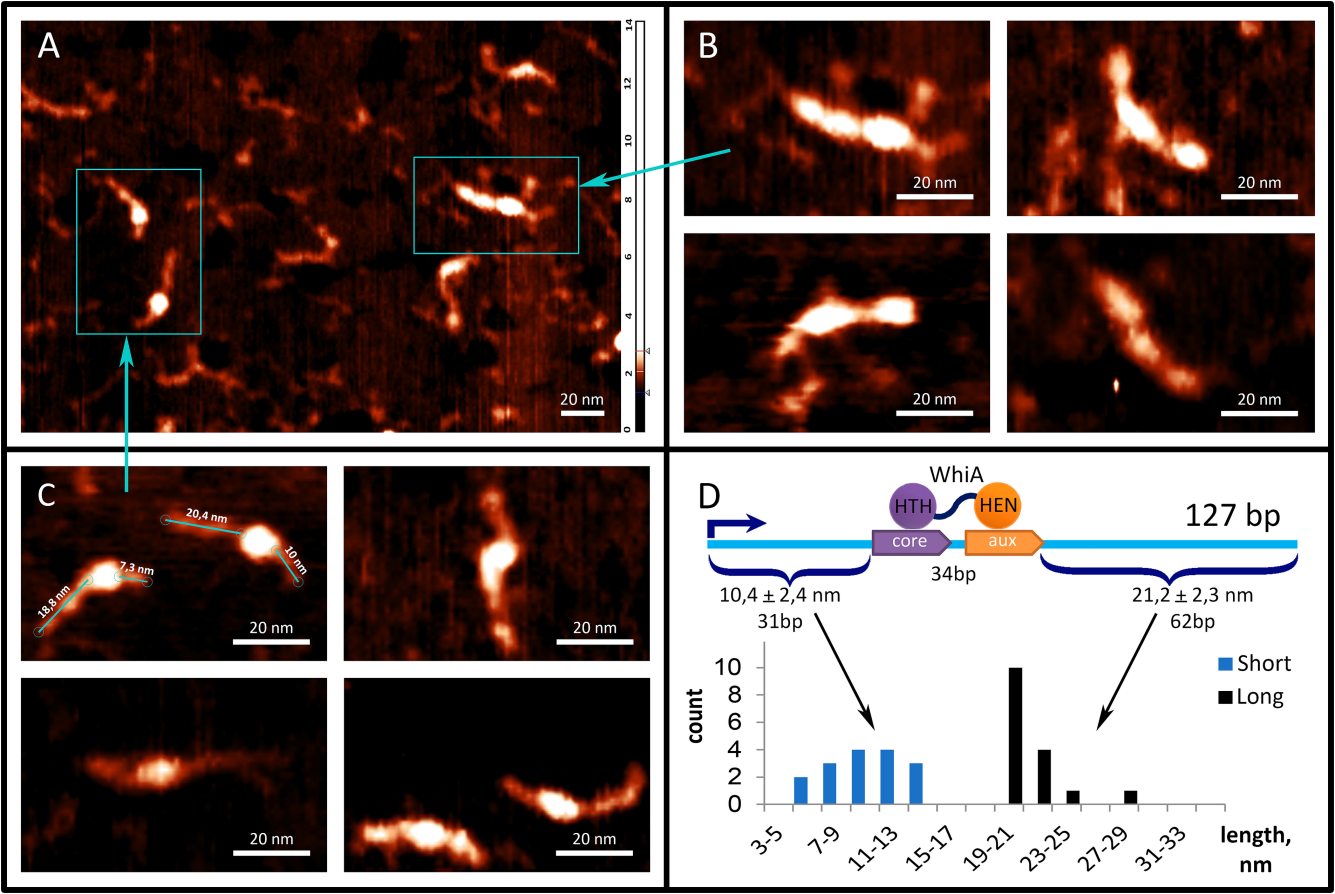
Mgal-WhiA interaction with *rpsJ* operon promoter. **(A–C)** AFM images of Mgal-WhiA with 127 b.p. DNA fragment corresponding to the *rpsJ* operon promoter. The fragment includes -10-box and WhiA binding site. The schematic representation of the DNA fragment and the WhiA binding site within it are shown on panel **(D)**. **(A)** The fragment of the AFM field of view containing different complexes of the DNA fragment with Mgal-WhiA. **(B)** The magnified view of multimeric complexes of Mgal-WhiA with the same oligonucleotide. In these complexes Mgal-WhiA shows non-specific DNA-binding activity. **(C)** The magnified view of monomeric complexes of Mgal-WhiA with the DNA fragment. The localization of Mgal-WhiA in these complexes is not random. The distribution of free DNA tails of these complexes is shown on panel **(D)**. **(D)** The position of Mgal-WhiA within the monomeric complexes corroborates the location of its binding site predicted from the multiple sequence alignment and observed in EMSA experiments with mutated oligonucleotides.

To further study the interaction between the Mgal-WhiA and *rpsJ* operon promoters, we used AFM (Figure 4). We used a fragment of the *M. gallisepticum rpsJ* operon promoter region, including the Mgal-WhiA binding site and the -10-box of the promoter. The total length of the DNA fragments was 127 b.p. The WhiA-binding site is located asymmetrically within the DNA fragment (Figure 4D). The structures observed by AFM confirmed the binding of Mgal-WhiA to a single locus with asymmetric localization within the fragment (Figure 4C). The length of the DNA strands protruding from the complex was 21.2 ± 2.3 and 10.4 ± 2.4 nm (Figure 4D). Assuming the average length of the nucleotide pair along the DNA (0.34 nm), the free strands consisted of 62 and 31 bp, respectively. These data corroborate the localization of the Mgal-WhiA binding site (Figure 4D). Thus, Mgal-WhiA occupies slightly more DNA than its conserved binding site. Mgal-WhiA additionally covers approximately 8 bp upstream and 6 bp downstream of the conserved recognition region, which results in a total 34 bp locus being covered by Mgal-WhiA. AFM showed non-specific binding of Mgal-WhiA to DNA as well (Figure 4B). We observed multiple proteins attached to the same DNA molecule including dimeric and trimeric complexes (Figure 4B). The non-specific DNA-binding activity of the Mgal-WhiA observed in EMSA and AFM experiments corroborates SRRF microscopy data, where Mgal-WhiA was found to be associated with genomic DNA. Thus, we conclude that Mgal-WhiA in certain concentration can occupy all available DNA via non-specific binding activity. We also conclude, that the dimeric complex observed with 40 b.p. oligonucleotide in EMSA experiments is a particular case of oligomeric binding observed with longer oligonucleotide in AFM experiments.

### 3.7 HTH domain of Mgal-WhiA specifically recognizes the core motif and is responsible for non-specific DNA binding

**Figure 5:**
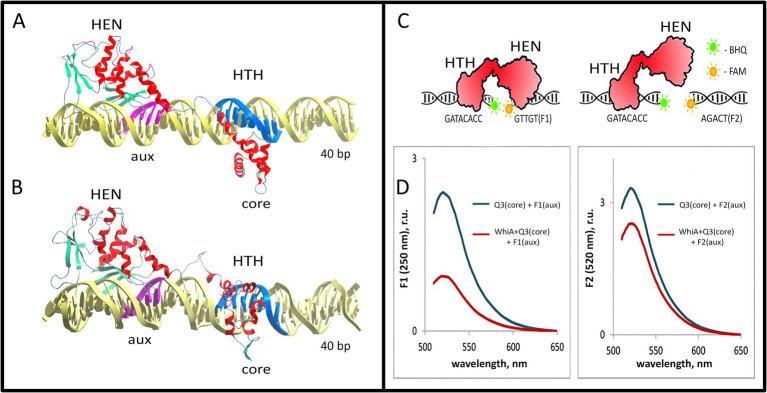
Model of the Mgal-WhiA complex with the *rpsJ* operon promoter. **(A,B)** The structure of the Mgal-WhiA complex with DNA at the beginning **(A)** and at the end **(B)** of the molecular dynamics simulation. The complex **(B)** demonstrates DNA duplex distortion during the MD simulation. **(C,D)** The validation of the complex structure using the FRET experiment. **(C)** The schematic representation of the FRET experiment. The binding site of Mgal-WhiA was split into two halves containing either core (Q3 oligonucleotide, BHQ1-labeled) or aux motif (F1 oligonucleotide, 6-FAM-labeled). The control oligonucleotide featured mutated aux motif (F2 oligonucleotide, 6-FAM-labeled). **(D)** The assembly of the complete complex (WhiA+core+aux) resulted in significant quenching of the 6-FAM fluorescence in comparison to the control (WhiA+core+control). Thus Mgal-WhiA specifically recognizes aux motif and binds DNA in a bipartite mode.

To further study the conformation of the Mgal-WhiA complex with the *rpsJ* promoter, we used docking and MD approach (see Methods section for details). We used the resolved 3D structure of WhiA from *Thermotoga maritima* 3hyi.1 for homology modeling. The same fragment of the *rpsJ* promoter used for the EMSA was used for modeling. The modeling was performed in two stages. After the initial docking (Supplementary structure S1), the stability and conformational dynamics of the complex were assayed by MD (Supplementary structure S2, see Methods section for more details). Modeling of the Mgal-WhiA interaction with DNA showed bipartite binding. HTH and HEN domains interacted with DNA at two loci corresponding to the core and auxiliary motifs, respectively (Figure 5; Supplementary Figures S12, S13).

The overall configuration of the DNA-binding interface of the Mgal-WhiA HTH domain was rather different from that of the HTH domain of an average transcription factor. It interacts with the major groove of DNA via two *α*-helices. One of the helices enters the major groove, similar to the other HTH-domains. At the same time, the second DNA-binding α-helix represented a part of the linker between the HTH and HEN domains. Both helices formed a pincers-like structure, which gripped the major groove from opposite directions. The structure somewhat resembled the DNA-binding region of the leucine-zipper TFs.

The HEN domain bound DNA via a *β*-sheet that entered the major groove of the DNA. The same mechanism for DNA binding has been described for homologous homing endonucleases. Our model shows that the HEN domain additionally covers approximately 7 bp downstream of the aux motif, possibly interacting with the whole DNA helix turn. This corroborates with the AFM data which showed that Mgal-WhiA covers more DNA than just a conserved motif. Nucleotides downstream of the aux motif interacted with the same β-sheet as the auxiliary motif, while their conservation was poor. This indicates that the HEN domain is capable of non-specific interactions with DNA. In addition, MD simulations demonstrated distortion of DNA upon interaction with Mgal-WhiA. This may indicate that Mgal-WhiA distorts or bends DNA.

To test the model of the bipartite interaction of Mgal-WhiA with DNA and to test its specificity for the aux motif, we designed a FRET-based experiment (Figures 5C,D). We split the wild-type binding site into two short oligonucleotides with either a core or aux site. The core oligonucleotide carried a BHQ1 quencher on its 3′ end, and the aux oligonucleotide carried 6-FAM on the 5′ end. As we demonstrated earlier, Mgal-WhiA primarily recognizes the core sequence that carries the quencher. Thus, binding of Mgal-WhiA to the core as a preferable target cannot result in 6-FAM quenching. The only complex structure that allows for efficient quenching is the simultaneous binding of both oligonucleotides to the same Mgal-WhiA molecule. The localization of the fluorophore and the quencher was designed to obtain maximal juxtaposition if the structure of the Mgal-WhiA complex with both oligonucleotides reconstituted the structure of the wild-type bipartite complex predicted in the MD simulation. A mutated aux oligonucleotide was used as a control. In corroboration with the MD model, we observed strong quenching in the case of the core/aux pair and significantly weaker quenching in the case of the core/control pair. Thus, we conclude that Mgal-WhiA indeed binds DNA in a bipartite mode, where the aux motif serves as a second specific binding site in addition to the core motif.

Another MD approach was used to identify the amino acids of the protein that interact with DNA. To assess the possibility of interaction between amino acid and nucleotide atoms, the following algorithm was applied. For each step of the MD trajectory, the distances between each pair of atoms of the current DNA site and the amino acids located in its vicinity were calculated. Hydrogen atoms were also included in this study. Then, a minimal distance was found within the array of trajectories. Contact was considered to exist if the distance did not exceed 3 Å. The model showed that the Mgal-WhiA contact areas were wider than just specific recognition sites. However, we further focused on the protein regions that interacted with the core and auxiliary motifs (Supplementary Figure S13). We studied the conservation of WhiA homologs in Mollicutes with respect to the predicted DNA recognition regions. The respective protein regions were among the most conserved, which corroborates their important role in WhiA function (Supplementary Figure S14).

**Figure 6:**
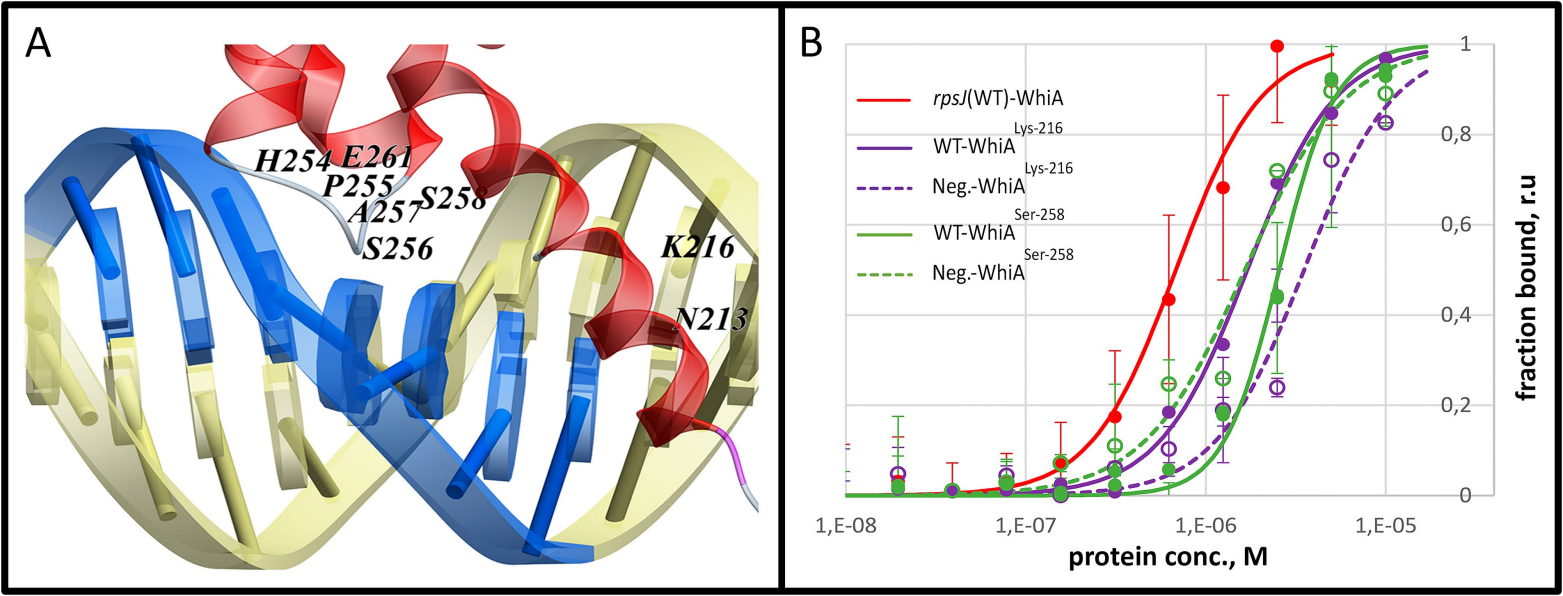
Mutational analysis of the Mgal-WhiA HTH domain. **(A)** Two amino acids (Lys-216 and Ser-256) that were identified in the molecular dynamics study as tightly interacting with the core motif were mutated to glycine. **(B)** MST analysis of the binding constant of the mutant WhiA forms. MST curve of wild-type Mgal-WhiA with rpsJ-WT oligonucleotide from Figure 3C (red line) is shown for comparison. The MST analysis included wild-type binding site oligonucleotide (WT) and the negative control oligonucleotide (Neg). The MST curves indicate the loss of specificity to the core motif for the both mutants.

To validate the MD modeling results, we performed point mutagenesis by substituting functional amino acids with glycine. The positions of the mutations were selected based on the MD data. The amino acids that formed the most contact with DNA within each contact zone were selected (Supplementary Figure S14). Based on this, we mutated Lys-216 (linker *α*-helix, Mgal-WhiA^Lys-216^) and Ser-258 (α-helix within the HTH domain, Mgal-WhiA^Ser-258^). Both mutations resulted in a loss of specificity for core motif recognition, while non-specific DNA-binding activity was retained (Figure 6). The Kd values of the mutant proteins were measured by MST. For Mgal-WhiA^Lys-216^ Kd for the WT oligonucleotide and the negative control were 1.7 ± 0.1 μM and 3.5 ± 0.9 μM, respectively. For Mgal-WhiA^Ser-258^ Kd it was 2.6 ± 0.2 μM and 1.7 ± 0.4 μM for both the WT and negative control oligonucleotides.

We propose that main contribution to both specific and non-specific DNA binding is paid by HTH domain. It happens due to the stronger interactions between HTH and core motif then between HEN and aux domain. This finding corroborates the data regarding the *S. coelicolor* core motif, where it is recognized by the HTH domain of WhiA and not by the HEN domain (Kaiser and Stoddard, 2011). Thus, we conclude that the ubiquitously conserved function of the HTH domain of WhiA across bacteria is the recognition of the core motif. The obtained data indicate that while the HEN domain can interact with DNA, its binding constant is significantly lower than that of the HTH domain. We also conclude that the oligomeric WhiA binding, observed on EMSA and AFM images, occurs via the non-specific DNA-binding activity of the HTH domain.

The modeling results corroborate the AFM observation, in that Mgal-WhiA binds along the DNA covering 34 b.p. The bipartite binding of the monomer completely sterically blocks the 40 b.p. oligonucleotide used for EMSA. Thus, we conclude that the observed dimeric complex is formed by two Mgal-WhiA molecules independently bound through the HTH domains to the same oligonucleotide.

### 3.8 The interaction of Mgal-WhiA with the auxiliary motif is crucial for transcriptional regulation

To confirm the role of the core motif and to elucidate the function of the auxiliary motif, we studied the transcriptional regulation by Mgal-WhiA in a reporter system. The previously obtained transcription start sites (TSS) maps for class Mollicutes representatives *M. gallisepticum* (Mazin et al., 2014), *Spiroplasma melliferum*, and *Acholeplasma laidlawii* (Fisunov et al., 2016b) were used as references to search for the *rpsJ* operon promoters within the Mollicutes. The TSS map for *M. gallisepticum* allowed us to identify and test the wild-type *rpsJ* operon promoter in a reporter system.

**Figure 7:**
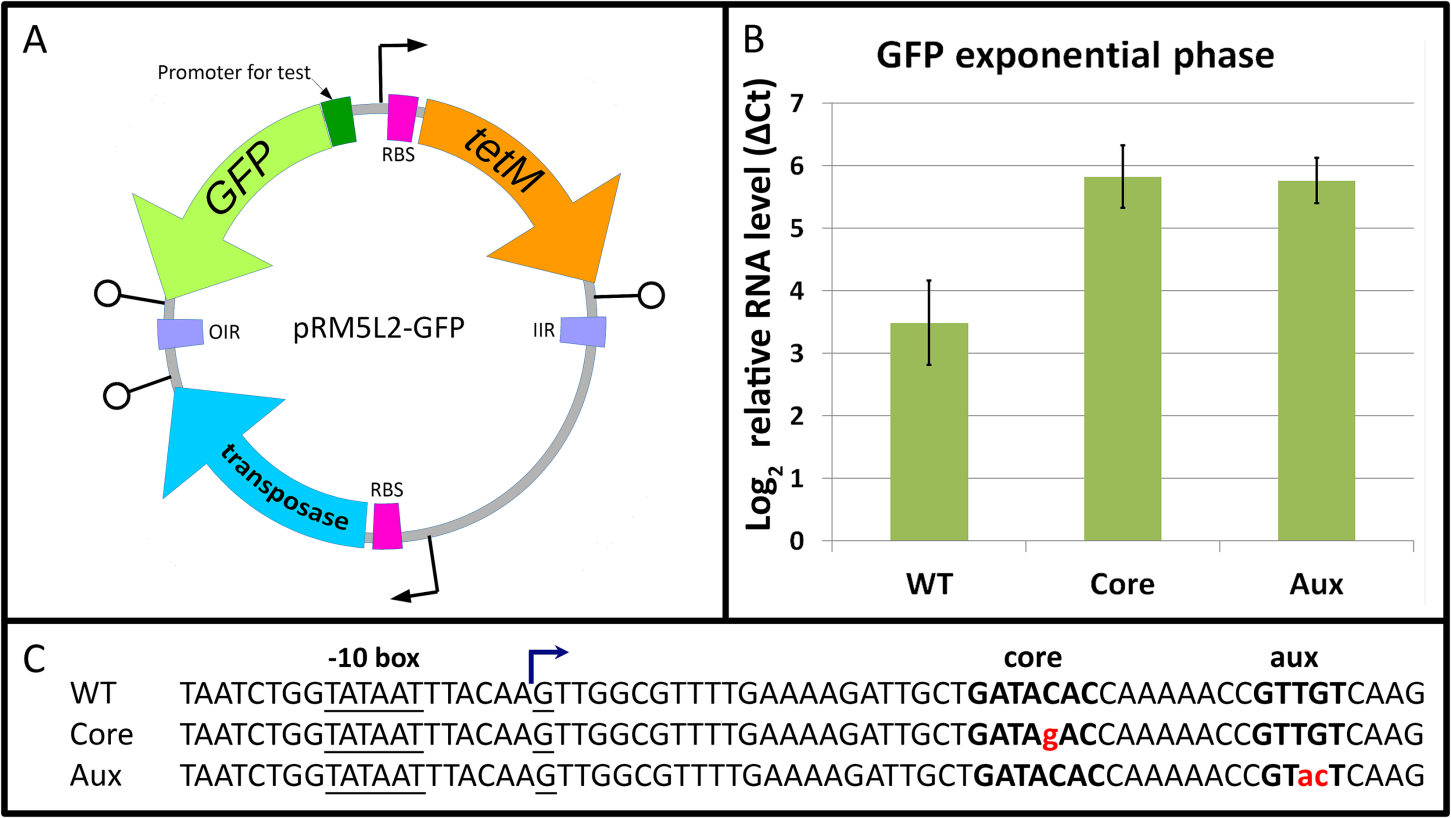
The role of core and auxiliary motifs in Mgal-WhiA repressor function. **(A)** The overview of the transposon vector used to test promoter activity. The translation of the transposase gene in *E. coli* is blocked by three nonsense codons, TGA, that are readthrough in mycoplasmas as Trp-codons. The sequence transferred to the genome is encompassed between OIR and IIR transposition motifs and integrates randomly. **(B)** Transcription activity of WT *rpsJ* promoter and *rpsJ* promoter with substitutions in core or auxiliary (aux) sequences. The level of *rpsJ* mRNA was measured using RT-qPCR relatively to enolase (*eno*) transcript. The substitutions in either core or aux motifs induce promoter activation relative to WT sequence. Bars indicate standard error. **(C)** The sequences of promoters used for testing. Mgal-WhiA binding site is highlighted in bold, substitutions are highlighted in red. TSS was identified in Mazin et al. (2014).

To test the function of Mgal-WhiA as a transcription factor, we fused the *rpsJ* promoter of *M. gallisepticum* with EGFP ORF (Supplementary Table S2) and tested the mRNA level rate in *M. gallisepticum* by real-time PCR. We used the WT Mgal-WhiA binding site, as well as its mutated variants with disrupted core or aux motifs. Since Eilers (2010) observed the effect of WhiA knockout in *M. pneumoniae* in the absence of *rpsJ* operon repression in the stationary phase, we used cells at both exponential and early stationary phases. However, we found that in *M. gallisepticum*, the wild-type binding site had a repressive effect on the promoter at the exponential phase already, while mutations of either core or aux motifs eliminated repression (Figure 7). Single point mutation within the binding site may not completely prevent WhiA binding. However, the observed trend shows that disruption of both motifs results in at least partial elimination of WhiA-induced repression. The latter indicates that WhiA has to bind at both core and aux motifs to perform the repressor function and confirms the results of MD and AFM. In the stationary phase, promoters carrying wild-type or mutated WhiA binding sites underwent strong repression (Supplementary Figure S15). The repression of the reporter at the stationary phase did not depend on the presence of the Mgal-WhiA binding site. It has been previously demonstrated that *M. gallisepticum* undergoes global gene repression in the stationary phase (Mazin et al., 2014), however the mechanism has not been clearly understood. Thus, we conclude that WhiA is indeed a transcriptional repressor of *rpsJ* operon, but the conditions that trigger de-repression of the *rpsJ* operon are different in *M. gallisepticum* and *M. pneumoniae*. We also conclude that interaction with the intact aux motif is crucial for WhiA repressor function.

Further, we studied the distribution of distances between TSS and WhiA binding sites within Mollicutes (Supplementary Figure S16). In the vast majority of Mollicutes, it was located significantly downstream of the TSS, forming two peaks: about 40 nt downstream of TSS, characteristic of acholeplasmas, and 25–30 nt downstream of TSS, characteristic of other species. In *M. gallisepticum*, the distance was 22 nt downstream to TSS. Thus, the aux motif is not a -35 box. The observed localization of the WhiA binding site leaves the possibility of transcriptional repression via the blocking of transcription elongation.

To investigate whether Mgal-WhiA impedes transcription elongation, we constructed a reporter system in which the Mgal-WhiA binding site (either wild-type or mutant) was located within the intergenic region between two genes coding for mMaple2 and YFP (Supplementary Table S2). The promoter was about a kilobase upstream of the Mgal-WhiA binding site. Insertion of the WhiA binding site affected the transcript level of the downstream gene, but the effect was very weak (Supplementary Figure S17). This effect was mediated solely by the core motif, regardless of the auxiliary motif. Thus, we conclude that Mgal-WhiA represses transcription initiation rather than elongation. One can speculate that Mgal-WhiA inhibits transcription initiation via local distortion of the DNA helix in the promoter region. We also hypothesized that the inhibition of transcription initiation may occur at the stage of abortive initiation, when RNA polymerase moves some nucleotides downstream from the TSS to bound WhiA.

### 3.9 HEN domain is responsible for ATP-modulated interaction with the auxiliary sequence

**Figure 8:**
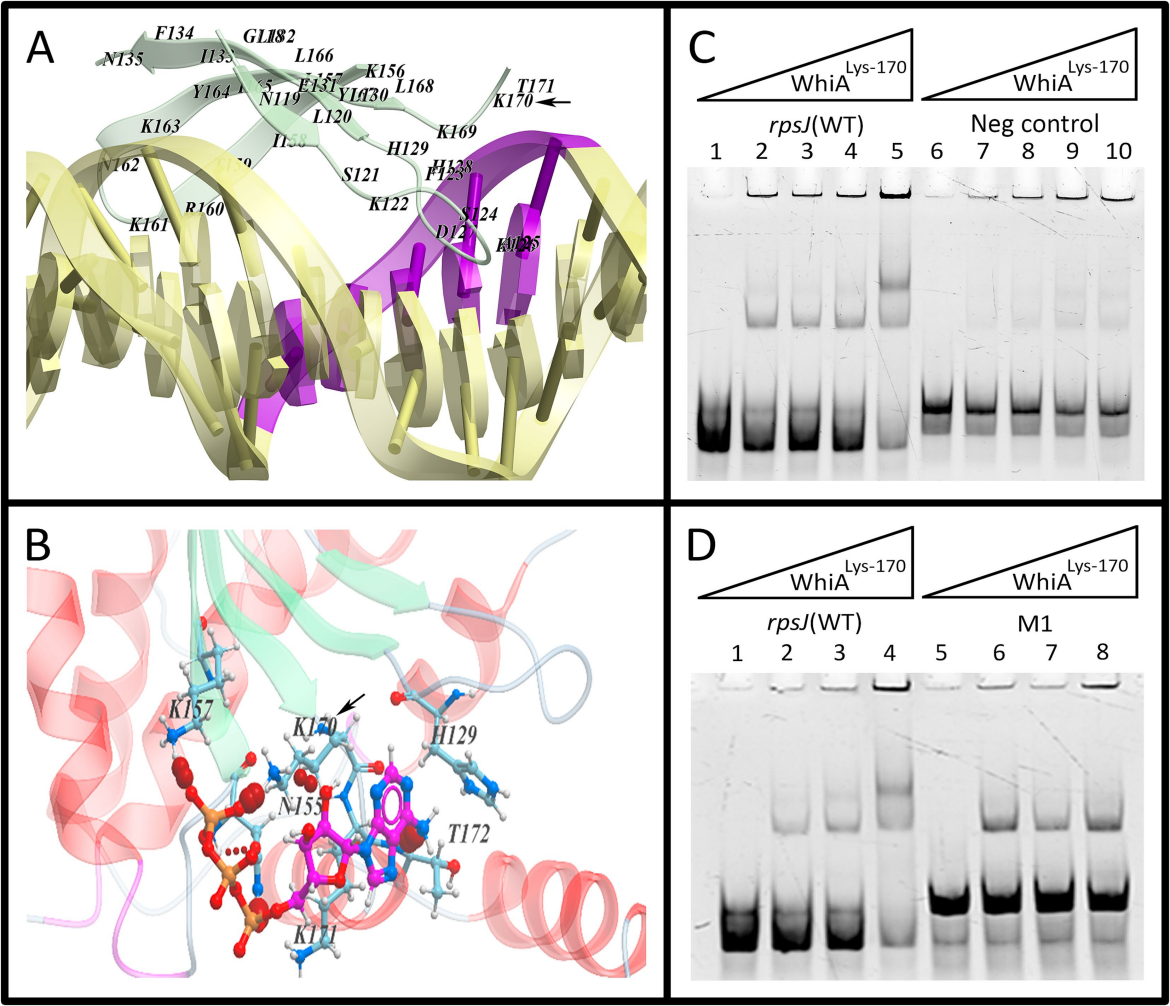
The effect of HEN-domain mutation on Mgal-WhiA regulation by ATP. **(A)** The HEN-domain interaction with aux motif in the molecular dynamics (MD) simulation. The simulation showed that Lys-170 tightly interacts with DNA. **(B)** MD simulation demonstrated that ATP-binding pocket resides in the vicinity of Lys-170 and this residue interacts with ATP as well. **(C,D)** EMSA of Lys-170 mutant with wild-type binding site oligonucleotide (WT), aux-mutated oligonucleotide (Mut1) and negative control oligonucleotide (Neg). Lys-170 mutant forms the dimeric complex more efficiently with WT oligonucleotide in comparizon to both Mut1 and negative control oligonucleotides, e.g., the presence of aux motif results in the more efficient formation of the dimeric complex. **(C)** Lanes: 1 – free rpsJ WT oligonucleotide 250 nM (same for all lanes), 2 – 44 nM Mgal-WhiA^Lys-170^, 3 – 88 nM Mgal-WhiA^Lys-170^, 4 – 177 nM Mgal-WhiA^Lys-170^, 5 – 354 nM Mgal-WhiA^Lys-170^, 6 – free negative control oligonucleotide 250 nM, 7 – 44 nM Mgal-WhiA^Lys-170^, 8 – 88 nM Mgal-WhiA^Lys-170^, 9 – 177 nM Mgal-WhiA^Lys-170^, 10 – 354 nM Mgal-WhiA^Lys-170^. **(D)** Lanes: 1 – free rpsJ WT oligonucleotide 250 nM, 2 – 88 nM Mgal-WhiA^Lys-170^, 3 – 177 nM Mgal-WhiA^Lys-170^, 4 – 354 nM Mgal-WhiA^Lys-170^, 5 – free Mut1 oligonucleotide, 6 – 88 nM Mgal-WhiA^Lys-170^, 7 – 177 nM Mgal-WhiA^Lys-170^, 8 – 354 nM Mgal-WhiA^Lys-170^.

We used MD simulations of ATP interaction with Mgal-WhiA (Figure 8; Supplementary Figure S18). The simulation revealed a set of possible ATP binding sites. Further we focused only on those located close to DNA-interacting surfaces. We hypothesized that ATP can sterically impede binding of at least one of the domains to DNA. The simulation identified an ATP-binding cavity within the HEN domain close to the aux-interacting half of the DNA-binding *β*-sheet. Additionally, a possible ATP binding site was identified within the HTH domain. Further we focused on these sites. The mutation of Lys-216, an amino acid residue that is involved in the contact with ATP within the HTH domain, happened according to the model described above. This mutation decreased the protein affinity and selectivity (Figure 6B; Supplementary Figure S19). The effect of ATP on Lys-216 mutant was not different from the effect on WT protein, e.g., ATP favored dimeric complex formation (Supplementary Figure S19). Thus, we conclude that even if ATP is able to bind to the HTH domain, it does not affect the ATP-dependent conformational changes from two-domain to single-domain binding. However, at the same time, it may contribute to ATP-mediated enhancement of the HTH-domain binding to the core sequence.

We proposed that ATP inhibits HEN-domain interactions with DNA by shielding its positive electrostatic charge. To test this hypothesis, we performed a mutational analysis of the HEN domain (Figure 8). The amino acid substitution was selected based on the results of MD simulation of the Mgal-WhiA interaction with both DNA and ATP. We mutated Lys-170 within the HEN domain (Mgal-WhiA^Lys-170^), which makes maximum contact with DNA and is located close to the ATP-binding cavity.

The DNA-binding activity of the Mgal-WhiA^Lys-170^ mutant was tested using EMSA. The effect of the Lys-170 mutation was the same as that of ATP (Supplementary Figure S9). It shifted equilibrium toward HEN-domain dissociation (dimeric complex) if the aux motif was present in the oligonucleotide. Hence, we conclude that the HEN-domain indeed interacts with the auxiliary motif, and this interaction is attenuated by ATP (and to a lesser extent by other NTPs).

MD simulation of the interaction of Mgal-WhiA with DNA showed that the protein distorts the DNA helix. We proposed that Mgal-WhiA bends DNA or traps DNA in the strained conformation by binding it with both domains simultaneously. In relaxed conformation Mgal-WhiA binds DNA only by the HTH-domain. The MD simulation also allows for the non-specific binding of the HEN-domain to DNA. At least half of the DNA-interacting β-sheet does not recognize any motif and the sequence downstream of the aux motif does not show any traces of conservation. Thus, we propose that the HEN-domain may either bind DNA non-specifically or recognize and grip the aux motif.

### 3.10 WhiA modulation imposes direct and indirect effects on the *Mycoplasma gallisepticum* proteome

To elucidate the role of WhiA in the global regulation of gene expression in *M. gallisepticum* we performed quantitative proteome study of WhiA-overexpressing strains, knockdown strains, control strains with dCas9 expression and the wild-type strain (Figure 1C; Supplementary Figure S20; Supplementary Table S5). Two strains and three biological replicates were used for each experiment.

First, we assayed the abundance of WhiA protein. WhiA abundance was the same in the dCas9 control and WT strain. In the overexpressing strain, WhiA was expressed two orders of magnitude more. In the knockdown strain, it was repressed by an order of magnitude compared to the control strain. Thus, we achieved a dynamic range of 10^3^-fold changes in the WhiA concentration in the cell. We then analyzed the abundance of dCas9 protein. This was comparable to the abundance of glycolytic enzymes, which are among the most expressed proteins in *M. gallisepticum*.

The proteins encoded in the WhiA target *rpsJ* operon demonstrated upregulation in the knockdown strain and showed barely detectable downregulation in the overexpressing strain. The observed proteomic changes corroborate the transcriptional changes: the suppression of *whiA* transcription resulted in approximately 3-fold upregulation of the *rpsJ* operon transcription and 1.4-fold median upregulation of the encoded proteins. In the overexpressing strains, the observed downregulation was negligible for the *rpsJ* operon-encoded proteins, and for the majority of them it was below the statistical significance cutoff.

In addition to the direct effect on *rpsJ* operon expression, the concentration perturbations of WhiA resulted in indirect effects on the proteome. The ribosome constituents and the enzymes involved in intermediary metabolism represent major fractions of the *M. gallisepticum* proteome in terms of both protein abundance and protein repertoire (Fisunov et al., 2011). Thus, we compared the systemic response of these groups of genes to WhiA changes and dCas9 expression as a control compared to the wild-type strain. Metabolic proteins showed no global response in any strain. In contrast, ribosomal proteins showed a systemic upregulation in the knockdown strain. Upregulation occurred independently of the genomic localization of the respective genes. The most upregulated ribosomal proteins encoded in separate operons included RpsI (1.5-fold), RpsO (1.4-fold), RpmB (1.4-fold), and RpmH (1.5-fold), which changed more or equally compared to the median of *rpsJ* operon-encoded proteins. We propose that this effect is mediated by an increase in the stability of the respective proteins through the more efficient assembly of ribosomes. At the same time, knockdown strains demonstrated upregulation of charepones ClpB and DnaK compared to the dCas9 control as well as for the WT strain. The upregulation of chaperones (1.6-fold for ClpB and 1.3-fold for DnaK) was comparable to their induction in sub-lethal heat stress (Butenko et al., 2017). The effect was observed solely in the knockdown strains and thus was not caused by high expression and possible aggregation of dCas9 or TetM protein, or the action of tetracycline itself. We hypothesize that the knockdown strains suffer from unfolded protein stress.

Another indirect effect was observed in the WhiA-overexpressing strains. They were found to be downregulated by more than 2-fold in the *oppA* operon, which encodes peptide transporter subunits OppA, GCW_02965, DppB, DppC, DppD, and DppF. The respective promoter lacks the WhiA-binding site as well as its core sequence. The mechanistic link between WhiA and peptide transporters is yet to be discovered.

## 4 Discussion

### 4.1 The model of Mgal-WhiA functioning *in vitro* and *in vivo*

**Figure 9:**
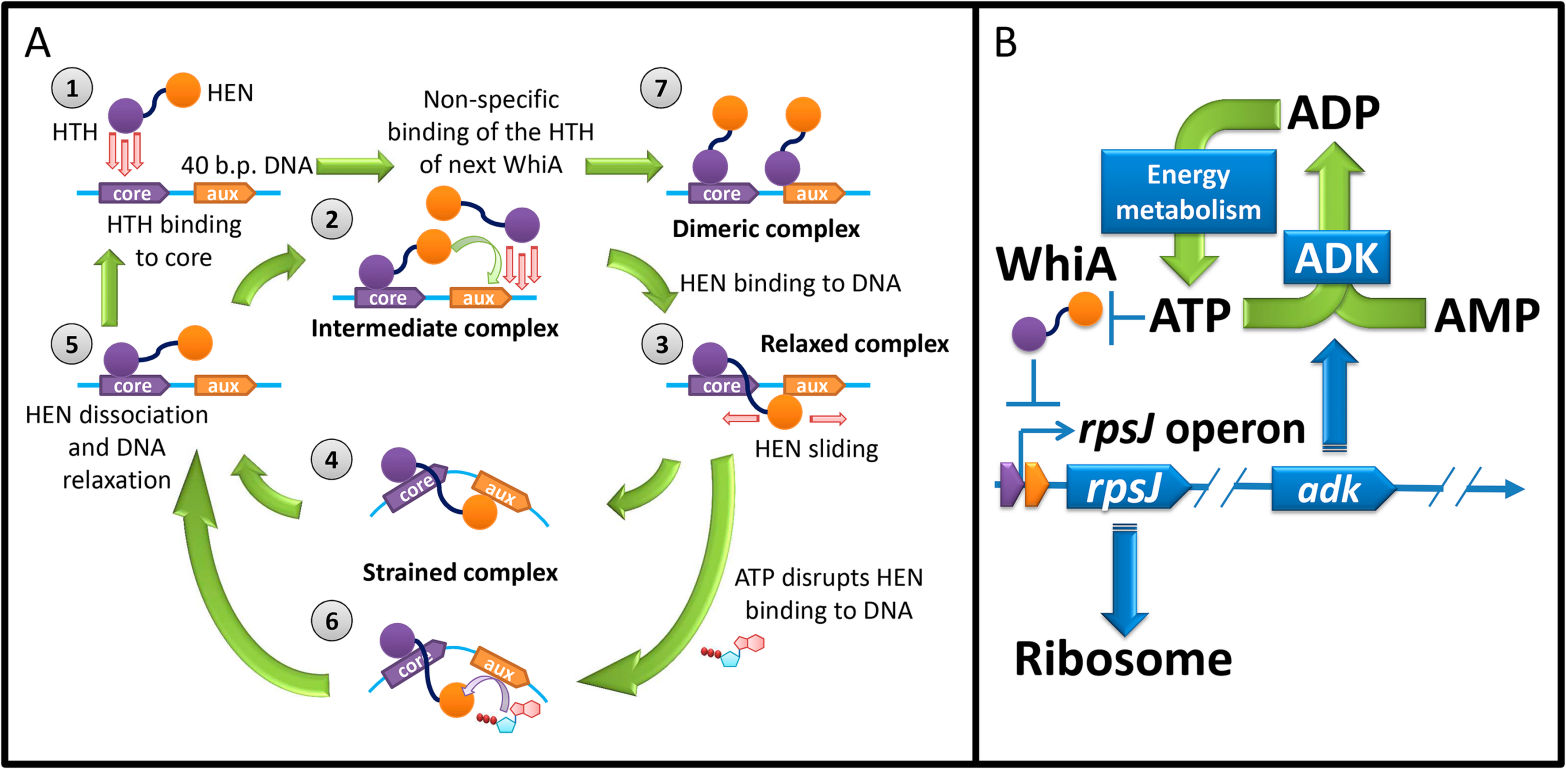
**(A)** The model of DNA-binding of Mgal-WhiA *in vitro* with 40 b.p. oligonucleotide. Stage 1 – HTH domain (violet) binding to the core motif. Stage 2 – The intermediate complex. HTH domain is bound to DNA, while the HEN domain (orange) protrudes into solution. If the DNA fragment is small (40 b.p. oligonucleotide) and the concentration of WhiA is high the competence between the HEN domain of the bound protein and the HTH domain of the second molecule from solution emerges. The binding of the second WhiA molecule occurs non-specifically. As a result if the HEN domain outcompetes, the complex proceeds to Stage 3. Otherwise the dimeric complex is formed (Stage 7). If the DNA fragment is large, the binding of WhiA molecules may proceed until all available space on DNA fragment is occupied. Stage 3 – The relaxed complex. The HEN domain weakly binds DNA and can probably slide along it. Stage 4 – The HEN-domain recognizes the auxiliary motif and locks DNA in a strained conformation. Stage 5 – The strained complex spontaneously dissociates due to the thermal fluctuations. The complex relaxes and proceeds to the intermediate stage. Stage 6 – ATP (and to lesser extent other NTPs) destabilizes the interaction of the HEN domain with the auxiliary sequence. Thus the half-life time of the strained complex decreases. **(B)** The model of the WhiA-mediated feedback loop between *rpsJ* operon expression and energy metabolism. *M. gallisepticum*. *rpsJ* operon encodes ribosomal proteins and adenylate kinase. The latter produces ADP from ATP and AMP and thus regulates the ADP pool available for phosphorylation. WhiA senses ATP concentration. At high ATP concentration it de-represses the *rpsJ* operon promoter, leading to the upregulation of ribosome proteins and adenylate kinase expression.

In summary, we proposed a model for the Mgal-WhiA functioning that explains its behavior both *in vitro* and *in vivo* (Figure 9). There is significant difference between *in vitro* experiments and *in vivo* functioning. First, the concentration of Mgal-WhiA *in vitro* is significantly higher than *in vivo*. Second, the DNA molecule *in vitro* is a relatively short ds-oligonucleotide, while *in vivo* it is genomic DNA. We propose that there is an equilibrium of three processes for the Mgal-WhiA with DNA: the specific binding of the HTH-domain to the core motif, the specific binding of the HEN-domain to the aux motif, and the non-specific binding of the HTH-domain to DNA.

*In vitro* (with 40-mer oligonucleotide) Mgal-WhiA initially binds to an oligonucleotide via the HTH domain (Figure 9A, Stage 1) and forms an intermediate complex (Figure 9A, Stage 2). Binding to the core motif occurs with a higher binding constant, but non-specific binding also occurs. The intermediate complex is bound only at the HTH domain, while the HEN domain protrudes into solution. The further binding may proceed two ways. HEN-domain of the bound WhiA binds at aux region and blocks the oligonucleotide or WhiA from the solution non-specifically binds the free space on the oligonucleotide. If the HEN-domain outcompetes, a monomeric complex is formed (Figure 9A, Stage 3). Alternatively, a dimeric complex emerges (Figure 9A, Stage 7). For the longer DNA molecules multiple proteins can bind DNA, producing multimeric complexes, which stoichiometry is limited only by DNA fragment length and the protein concentration. We propose that *in vivo* the competence of different Mgal-WhiA molecules for the binding to adjacent DNA loci is negligible, since its concentration *in vivo* is significantly lower than *in vitro*. *In vivo* Mgal-WhiA just binds core motif by HTH-domain and auxiliary motif by HEN-domain without any competence.

We propose that upon initial DNA binding, the HEN domain can slide along the DNA (relaxed conformation). If the auxiliary motif is present in DNA, the HEN domain further binds more tightly capturing DNA in a strained bent conformation (Figure 9A, Stage 4). HEN domain can dissociate from auxiliary motif through thermal fluctuations (Figure 9A, Stage 5). Subsequently, Mgal-WhiA proceeds to the intermediate complex when the HEN domain can reattempt to bind DNA.

The binding constant of the HTH domain is higher than that of the HEN domain; thus, the measurable Kd for Mgal-WhiA does not depend on the presence of an auxiliary motif, but crucially depends on the presence of the core motif. At the same time, the binding of Mgal-WhiA to both sequences was demonstrated in FRET experiments. We propose that the binding of the HTH domain to DNA drastically increases its local concentration, thus making the HEN-domain binding efficient. In the absence of auxiliary motif, the complex stacks on the relaxed stage (Figure 9A, Stage 3) and do not proceed to the strained complex stage (Figure 9A, Stage 4). *In vitro,* the deletion of the auxiliary motif from the 40 b.p. oligonucleotide extends the half-life of the bipartite-bound monomer by stacking in the relaxed complex stage and decreases the oligonucleotide accessibility for the second Mgal-WhiA molecule. We propose that *in vivo* the deletion of the auxiliary motif from the binding site makes Mgal-WhiA unable to grip DNA properly resulting in the loss of transcriptional repression.

ATP disrupts the interaction between the HEN domain and the auxiliary motif. This leads to the release of the HEN-domain from the auxiliary motif and leaves Mgal-WhiA bound only by the HTH-domain to the core motif (Figure 9A, Stage 6). *In vitro* it results in the equilibrium shift from the monomeric to the dimeric complex. We propose that *in vivo* Mgal-WhiA just releases the auxiliary motif, which is sufficient to de-repress a target promoter.

The bipartite binding of Mgal-WhiA to DNA in cis-conformation can provide grounds for the proposal that it may bind distant chromosome loci that contain core and aux motifs. The ability of Mgal-WhiA to bind two oligonucleotides that are not covalently linked supports this hypothesis. This binding in the trans-conformation may aid the formation of chromosomal loops. However, it seems more probable that Mgal-WhiA prefers binding in the cis-conformation to long DNA chains.

### 4.2 The function of WhiA for the proteome coordination in mycoplasmas

The data obtained on the role of WhiA in transcription regulation in *M. gallisepticum* and *M. pneumoniae* are different. In this study, we demonstrated that WhiA imposes repressor activity during the exponential growth phase. It has been demonstrated that in *M. pneumoniae* WhiA acts as a transcriptional repressor in the stationary phase (Eilers, 2010). We propose that this may be due to differences in the coordination of gene expression and energy metabolism in these species. The observed doubling time for *M. gallisepticum* culture was approximately 4 h (Gorbachev et al., 2013), while that for *M. pneumoniae* was approximately 20 h (Wodke et al., 2013). Thus, the growth rate of *M. gallisepticum* is significantly higher than that of *M. pneumoniae*, while the overall physiology and genome organization are very similar. Interestingly, the theoretically predicted growth rate for *M. pneumoniae* was within 2–4 h (Wodke et al., 2013), which is the same as that for *M. gallisepticum*. We propose that in *M. gallisepticum*, the obtained ATP immediately enters the synthesis of new biomass (RNA and protein). Thus, its intracellular levels remain permanently low. In *M. pneumoniae*, a slower growth rate favors the accumulation of ATP until the energy sources remain available, for example, during exponential growth. If this hypothesis is true, *M. gallisepticum* demonstrates a growth rate close to the maximum. In addition, the growth rate of *M. pneumoniae* is limited by factors other than the rate of energy metabolism. Hence, the ability of WhiA to repress the *rpsJ* operon is conserved, but from a physiological point of view, this depends on the lifestyle of a particular organism. WhiA knockdown in *M. gallisepticum* impacts the growth restart after the growth stop. However we did not observe a condition when *rpsJ* operon is de-repressed. We hypothesize that in *M. gallisepticum rpsJ* operon de-repression may occur at a particular stage of cell cycle and is triggered by the internal rather than the external stimuli. Key internal stimulus may represent intracellular ATP concentration oscillations. Since *M. gallisepticum* cells in culture are not synchronized the transient de-repression events in a small cell fraction are not detectable. The proposed regulatory circuit of WhiA in mycoplasmas is schematically summarized in Figure 9B.

The observed upregulation of chaperones in the *whiA*-knockdown strains led us to conclude that they suffer from unfolded protein stress. No such effect was observed for any of the overexpressing strains (WhiA and dCas9). Thus, unfolded protein stress is not induced by aggregation of overexpressed proteins. At the same time, we observed systemic upregulation of ribosomal constituents in the knockdown strains. We propose that the observed unfolded protein stress is the indirect effect of the loss of synchronization between ribosome production and the rest of the metabolic machinery. As demonstrated above, WhiA constitutively represses the *rpsJ* operon. The loss of WhiA function results in a misbalance in the cellular proteome. Therefore, we propose that WhiA is a sensor of internal clues rather than external stimuli. The major role of WhiA is the sensing of stochastic perturbations of the metabolic fluxes and countering chaos propagation that can arise from a misbalance between different biochemical processes.

### 4.3 Study limitations and future directions

In the current work we used *in vitro* models and laboratory growth conditions of *M. gallisepticum*. It remains unknown which natural conditions induce WhiA-mediated *rpsJ* operon upregulation. We can only conclude that they are different from the ones of *M. pneumoniae*. One can speculate that WhiA is involved in the continuous attenuation of ribosomal proteins production in response to fortuitous perturbations of ribosomal proteins amount in cell due to the gene expression noise. Thus its functional remains undiscovered on the cells’ population level. Thus single-cell approach has to be used to further discover WhiA function.

## Data Availability

The datasets presented in this study can be found in online repositories. The names of the repository/repositories and accession number(s) can be found in the article/Supplementary material.

## Footnotes

^1^ http://www.ebi.ac.uk/pride/archive/projects/PXD026928
