# Supplementary material for: WhiA transcription factor provides feedback loop between translation and energy production in a genome-reduced bacterium
Source: Front Microbiol. 2024 Dec 23;15:1504418. doi: 10.3389/fmicb.2024.1504418 (PMC11701221; doi:10.3389/fmicb.2024.1504418)
Supplement: Supplementary file 1 [file Data_Sheet_1.PDF]

Supplementary figures

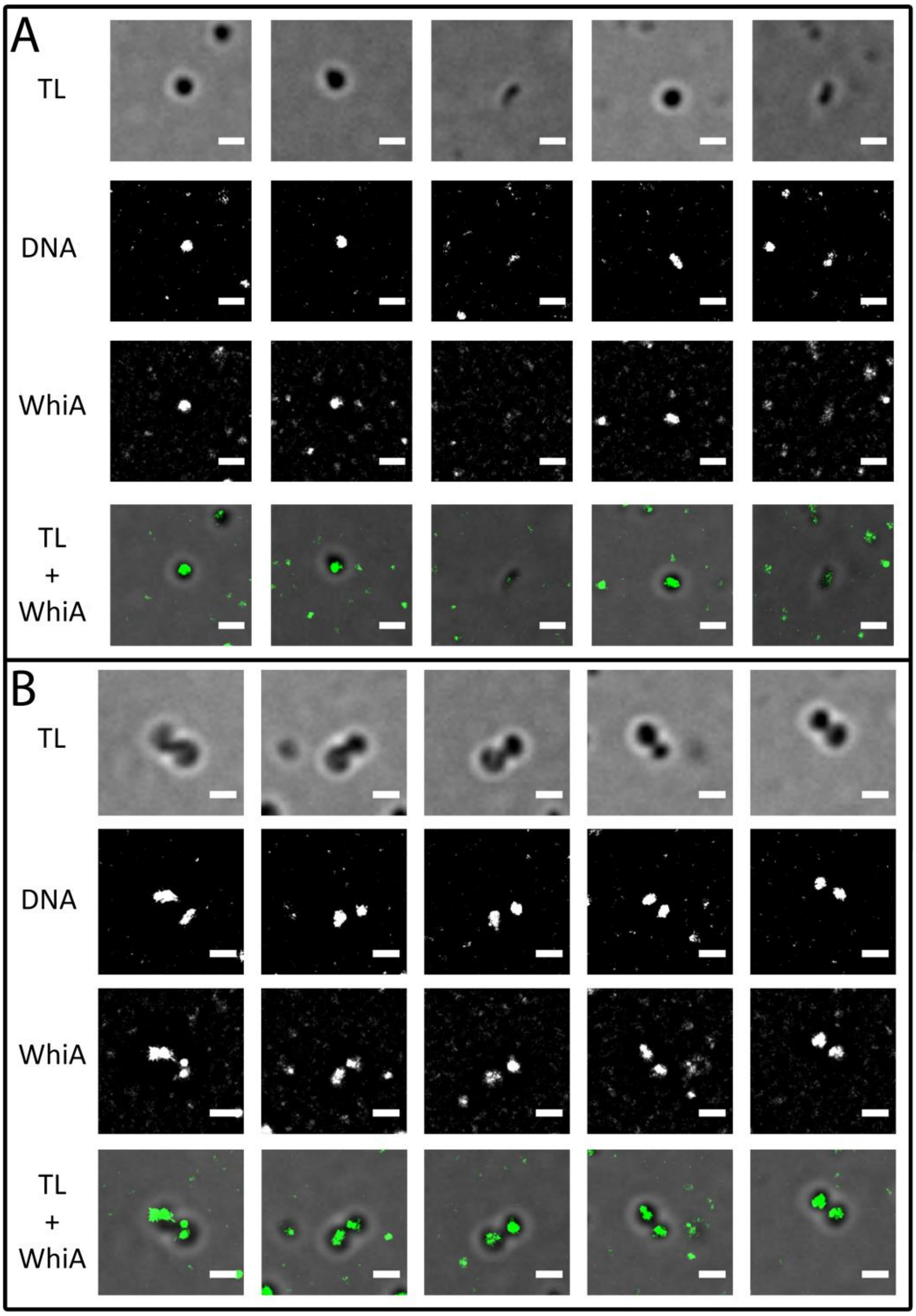

Supplementary figure S1. The distribution of WhiA protein and genomic DNA in living *M. gallisepticum* cells. WhiA protein from *M. gallisepticum* was fused with mMaple2 protein and expressed from transposon vector with strong promoter. The super-resolution microscopic images were obtained using SRRF method. Bar = 1 µm. TL – phase-contrast transmission light images of the living *M. gallisepticum* cells, DNA – SRRF images of the genomic DNA labeled with 5-SiR dye, WhiA – SRRF images of the *M. gallisepticum* WhiA protein labeled with mMaple2 fluorescent protein fusion. TL+WhiA – the superposition of TL and WhiA images. **A** – Single cells. **B** – Pairs of cells that, probably, undergo cytokinesis.

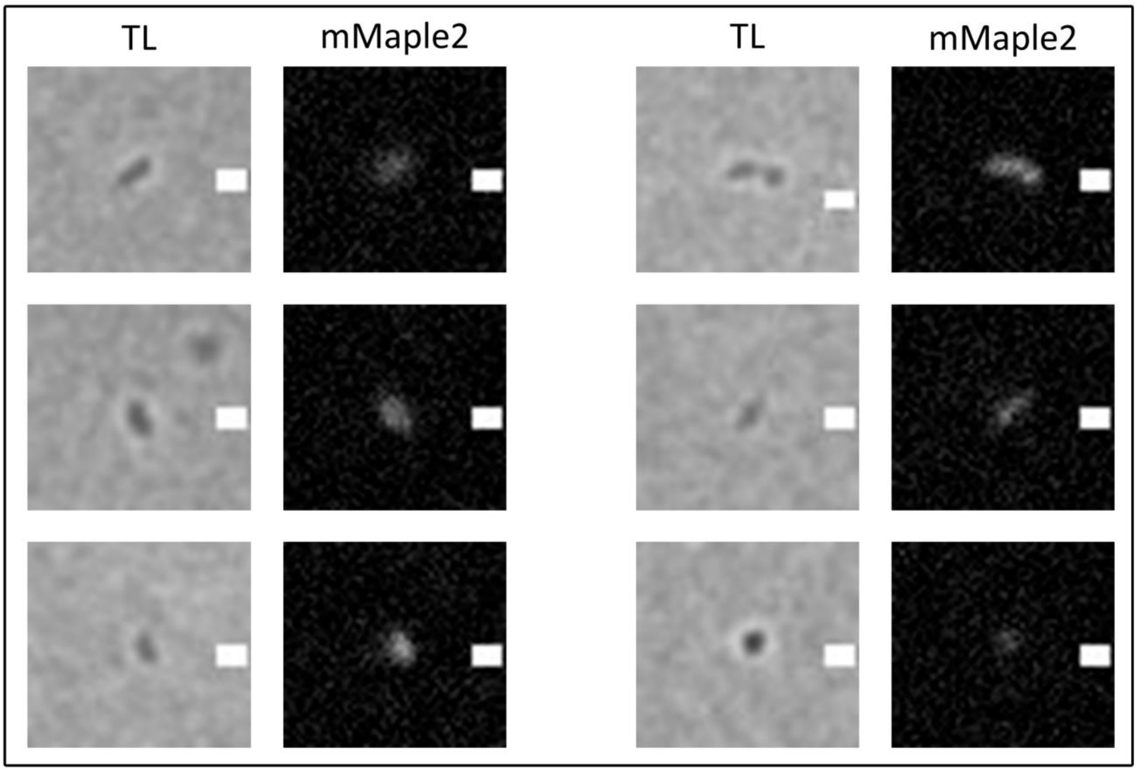

Supplementary figure S2. The distribution of mMaple2 protein expressed from transposon vector with strong promoter (same as on Figure S1) in living *M. gallisepticum* cells. The super-resolution microscopic images were obtained using SRRF method. Bar = 1 µm. mMaple2 expressing strain was used as a control of the fluorescence distribution. TL – phase-contrast transmission light images of the living *M. gallisepticum* cells, mMaple2 – SRRF images of the mMaple2 fluorescent protein.

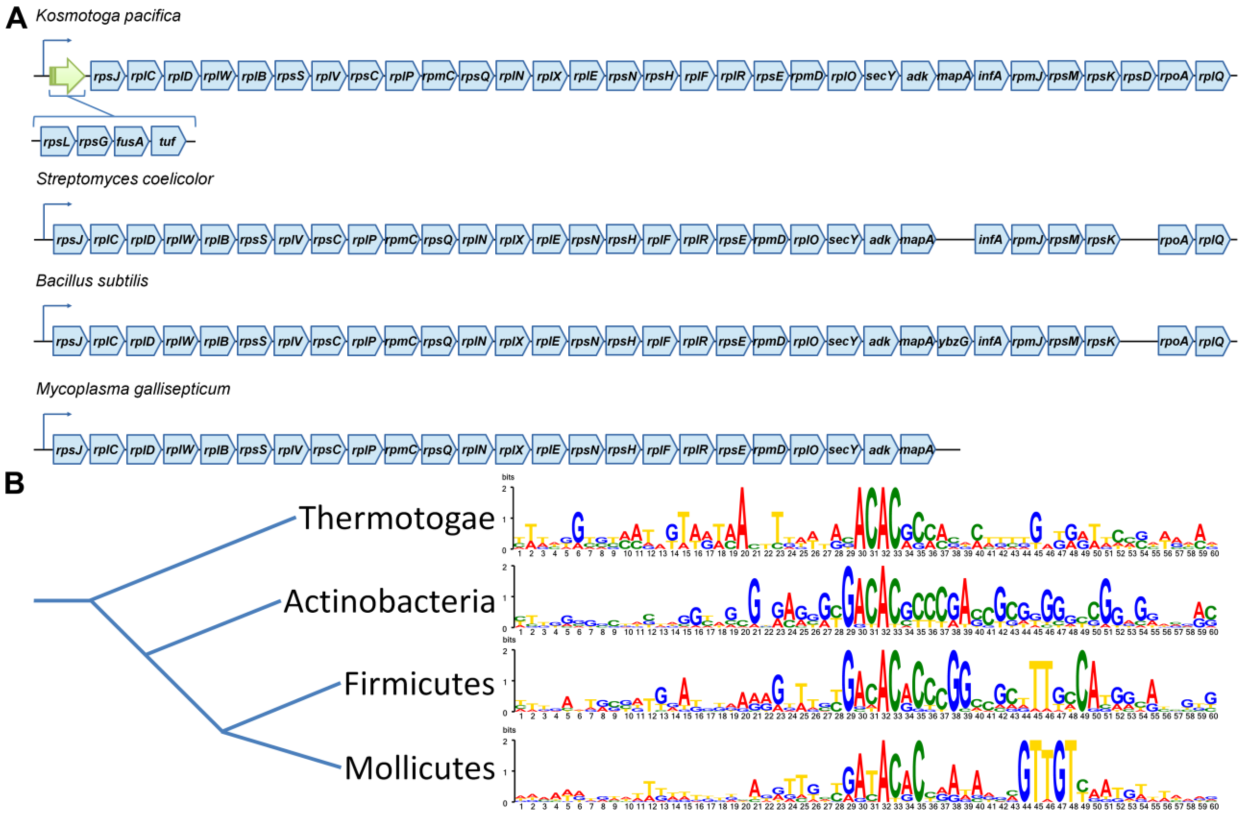

Supplementary figure S3. Conservation of WhiA binding site and the target *rpsJ* operon in Bacteria. A – The structure of *rpsJ* operons of bacteria from different phylogenetic clades, which features *whiA* gene. Adenylate kinase gene is a conserved member of the operon in distant bacterial clades. **B** – Conserved motifs in the *rpsJ* operon promoters of bacteria from different phylogenetic clades that correspond to WhiA binding site. The universal core motif is GAYACRCY, where Y is a pyrimidine (C or T) and R is a purine (A or G). There is also clade-specific auxiliary motif downstream (GTTGT in Mollicutes).

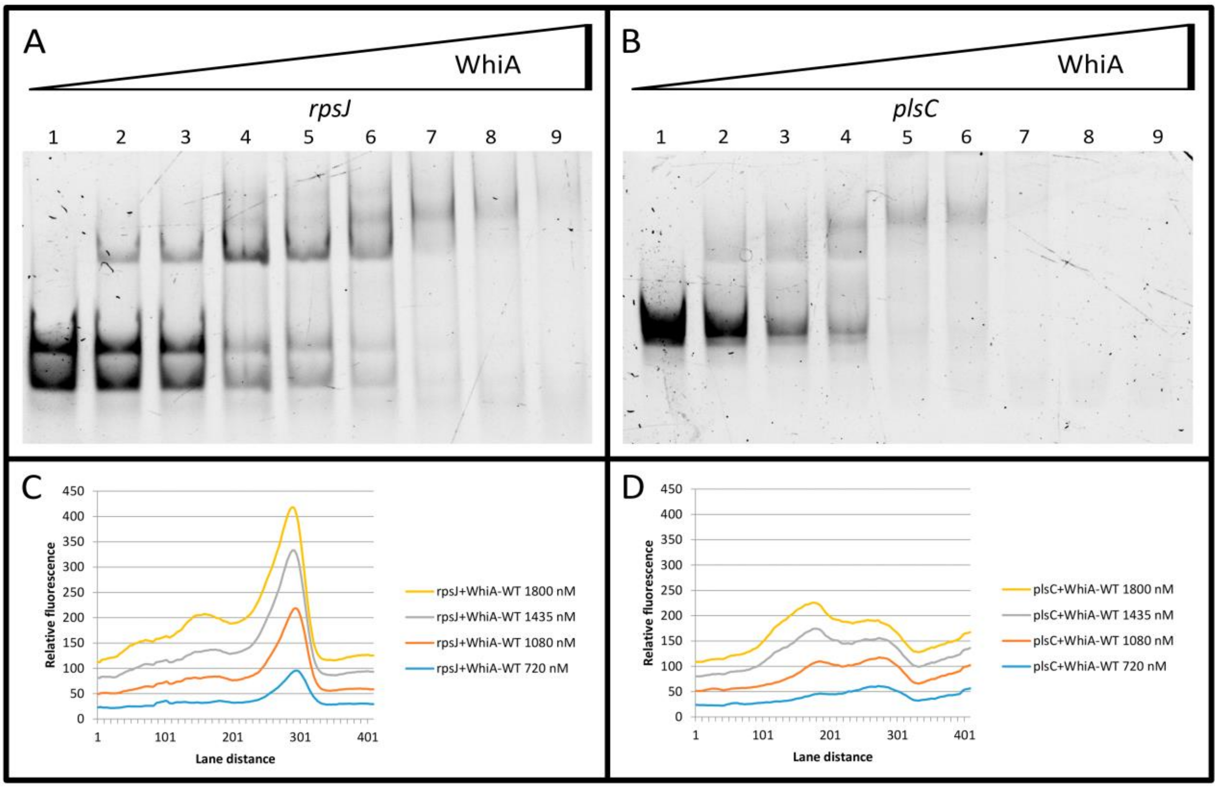

Supplementary figure S4. EMSA titration of Mgal-WhiA with WT-binding site from *rpsJ* promoter and with negative control (the fragment of *plsC* promoter). A – EMSA titration of Mgal-WhiA with *rpsJ* WT oligonucleotide. The following amounts of WhiA were used: 0 nM (1), 360 nM (2), 720 nM (3), 1080 nM (4), 1435 nM (5), 1800 nM (6), 2160 nM (7), 2520 nM (8), 2880 nM (9). **B** - EMSA titration of Mgal-WhiA with negative control (*plsC* promoter fragment) oligonucleotide. **C** – Densitometry plots of EMSA WhiA titration from **A** (*rpsJ* WT). Only DNA-protein complexes are presented on the plot. Unbound DNA not shown. **D** – Densitometry plots of EMSA WhiA titration from **B** (negative control).

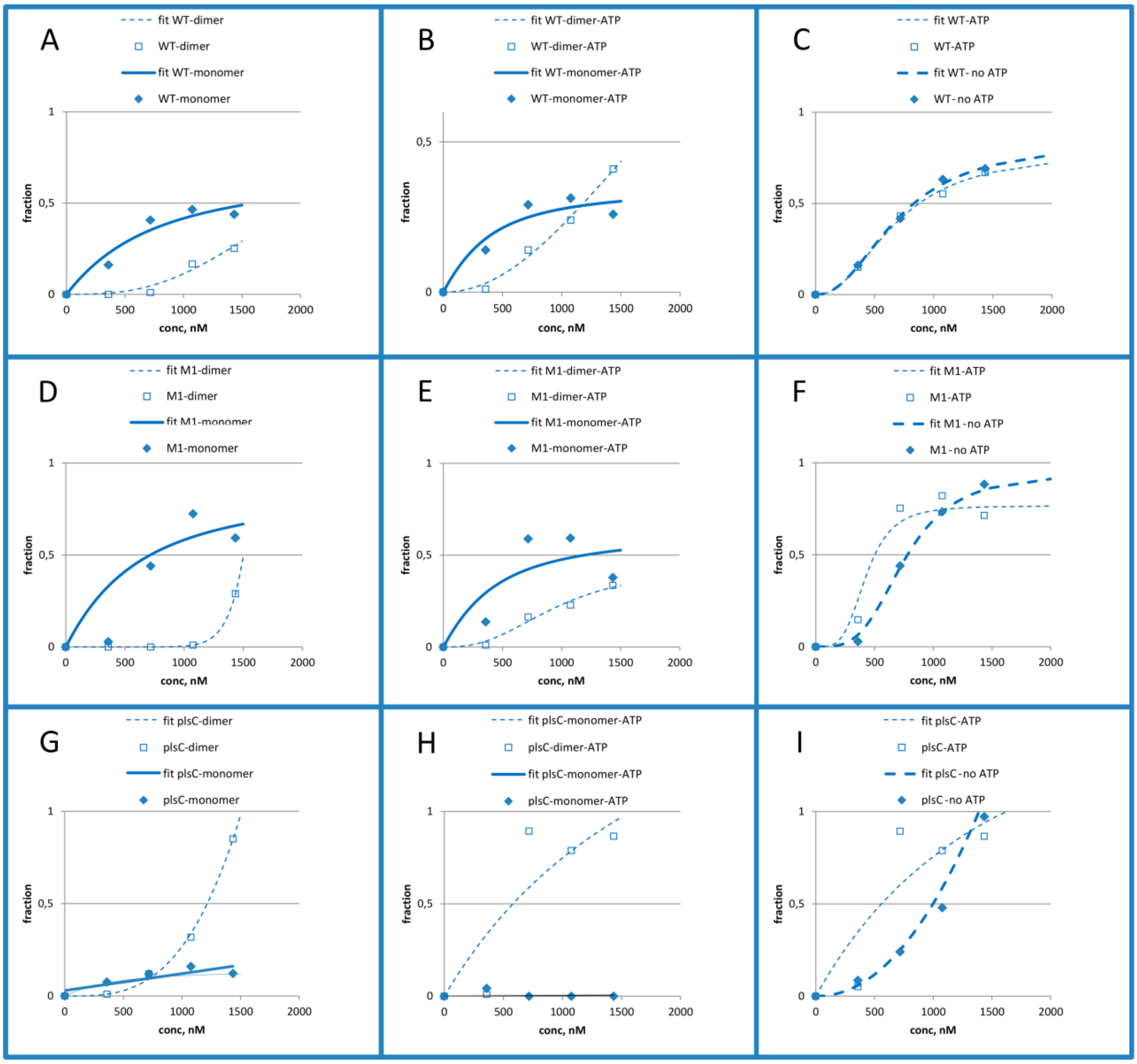

Supplementary figure S5. WhiA binding curves obtained from EMSA images and approximated by Hill equation. Dots represent the experimental data; curves show Hill equation approximation of the experimental data. ATP concentration in all samples supplied with ATP is 1 mM. Oligonucleotide concentration is 250 nM in each sample. **A-C** – EMSA of Mgal-WhiA with the wild-type (WT) binding site from *rpsJ* operon promoter of *M. gallisepticum*. **A** – Binding in absence of ATP, the contributions of the dimeric and monomeric complexes are calculated separately; **B** – Binding in presence of ATP, the contributions of the dimeric and monomeric complexes are calculated separately; **C** – The comparison of total binding of Mgal-WhiA in presence and in absence of ATP. **D-F** – EMSA of Mgal-WhiA with the binding site with the auxiliary motif disrupted by mutation. **D** – Binding in absence of ATP, monomeric and dimeric binding; **E** – Binding in presence of ATP, monomeric and dimeric binding; **F** – Total binding in presence and in absence of ATP. **G-H** – EMSA of Mgal-WhiA with the control oligonucleotide without either core or auxiliary motifs. **G** – Binding in absence of ATP, monomeric and dimeric binding; **H** – Binding in presence of ATP, monomeric and dimeric binding; **I** – Total binding in presence and in absence of ATP.

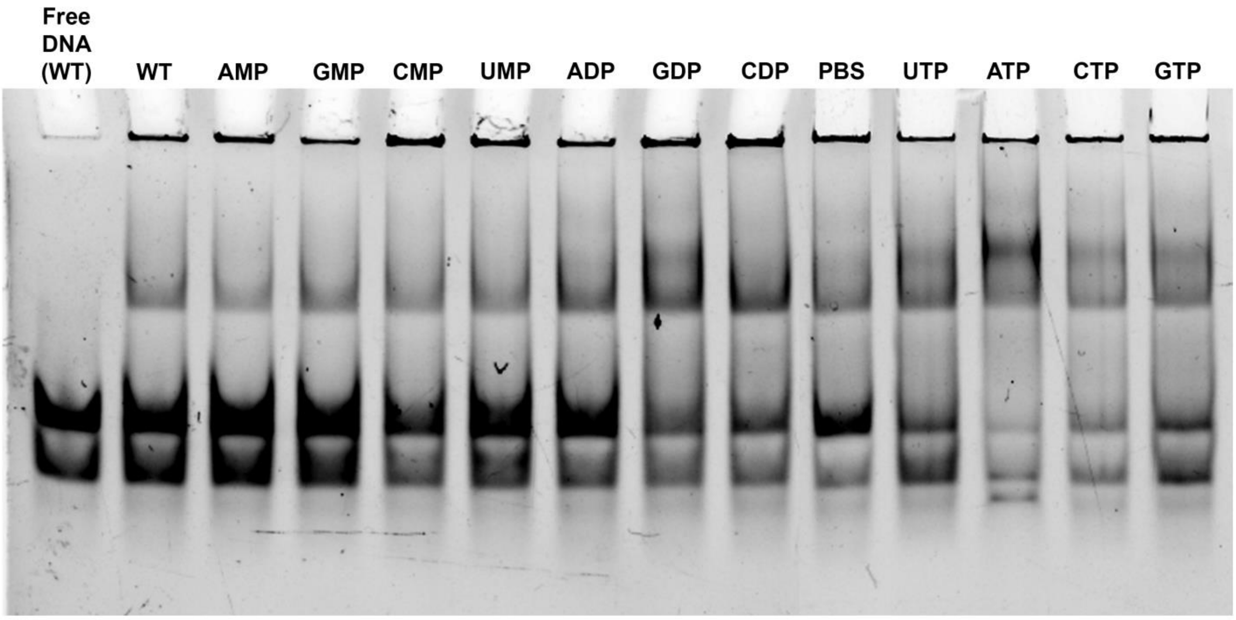

Supplementary figure S6. EMSA of Mgal-WhiA with wild-type binding site supplied with different nucleotides. The concentration of nucleotides was 1 mM for each reaction. EMSA with 1xPBS buffer is shown as a control for phosphate. Further we studied the effect of simultaneous presence of ADP and ATP in the reaction mixture, since both nucleotides are abundant in cell (Figure S8).

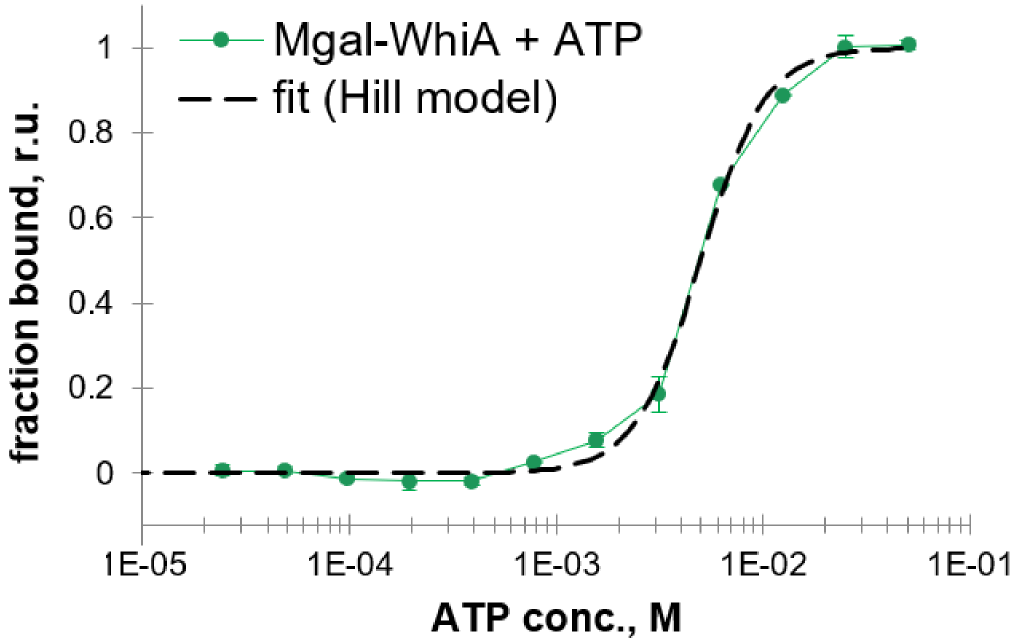

Supplementary figure S7. MST curve of Mgal-WhiA binding with ATP.

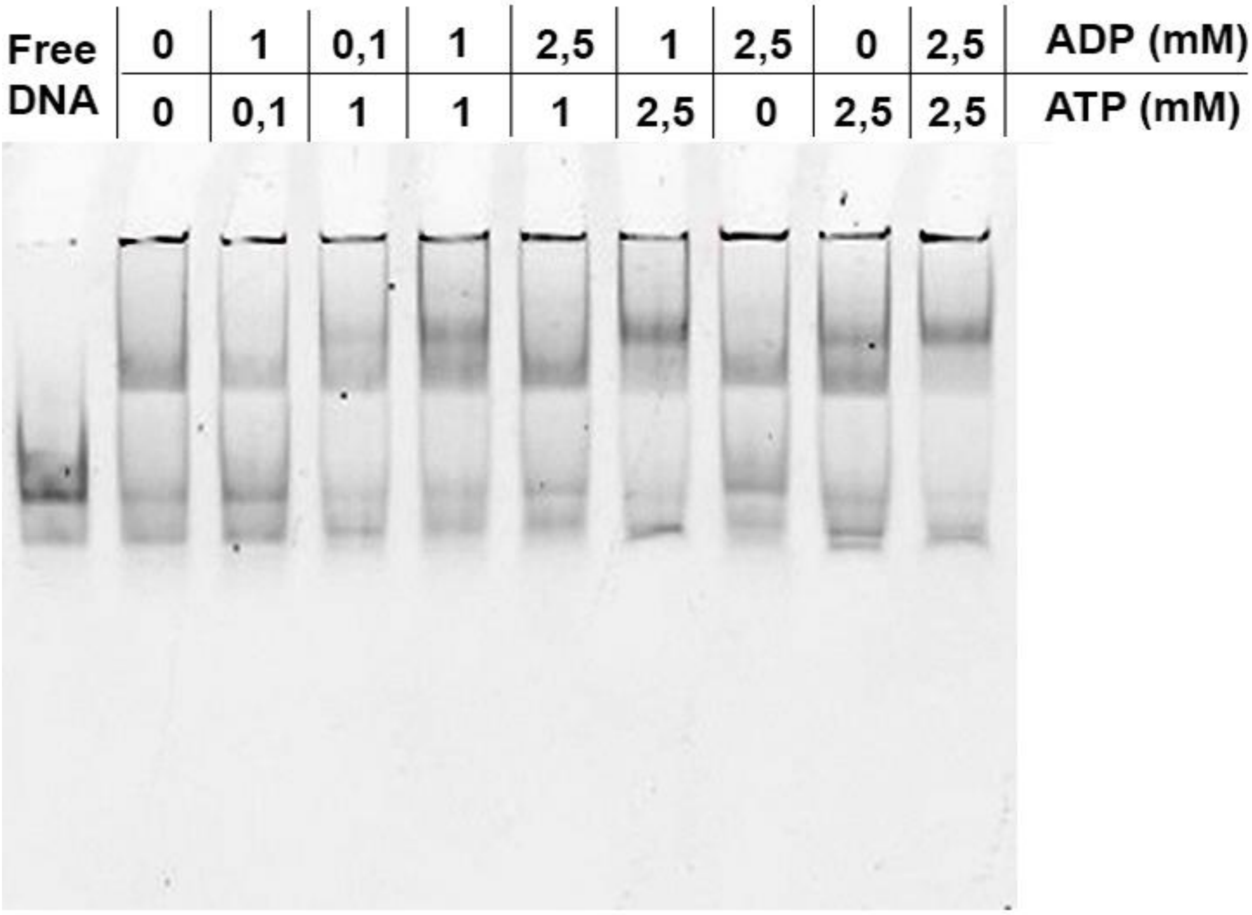

Supplementary figure S8. EMSA of Mgal-WhiA with the WT oligonucleotide supplied with ADP and ATP at different proportions. The main contribution to the monomer to dimer equilibrium shift is paid by ATP rather than ADP.

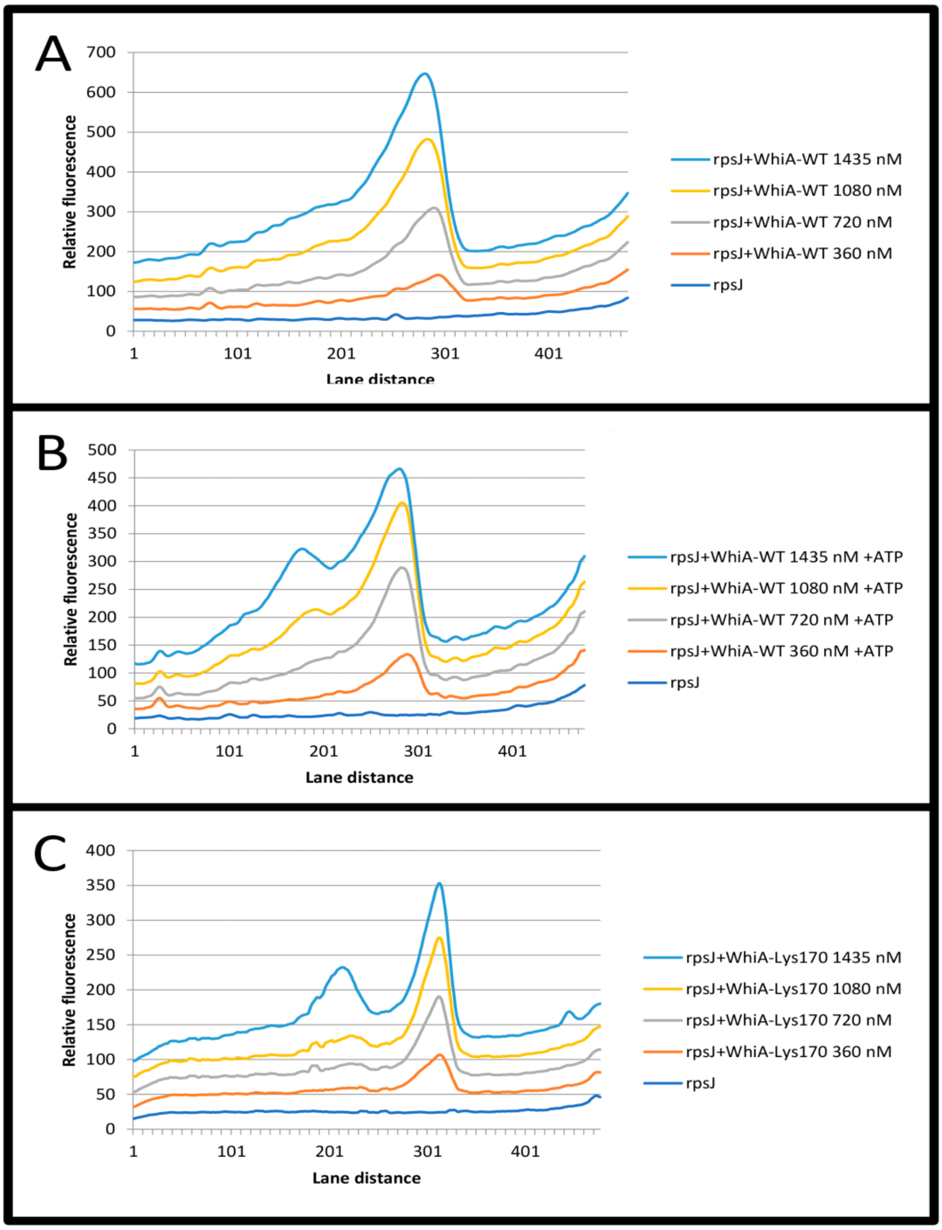

Supplementary Figure S9. **EMSA titration of Mgal-WhiA complex with *rpsJ* WT oligonucleotide represented as densitometry plots.** Area of non-bound DNA is not shown. Lane start is on the left. A – Complex of WT Mgal-WhiA with *rpsJ* WT oligonucleotide. B – Complex of WT Mgal-WhiA with *rpsJ* WT oligonucleotide in the presence of 1 mM ATP. C – Complex of Mgal-WhiA^{Lys170} mutant with *rpsJ* WT oligonucleotide.

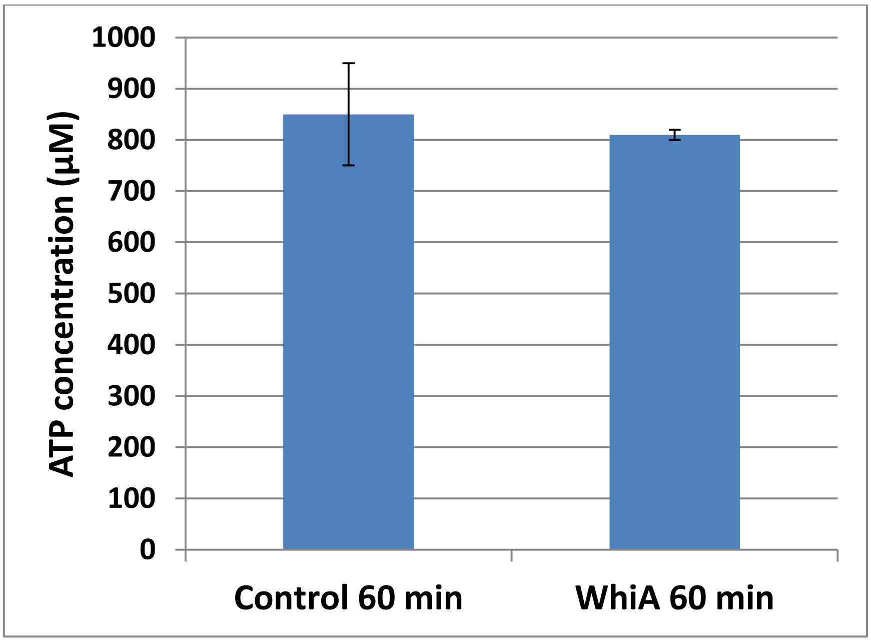

Supplementary figure S10. Luciferase assay for putative ATPase activity of WhiA.

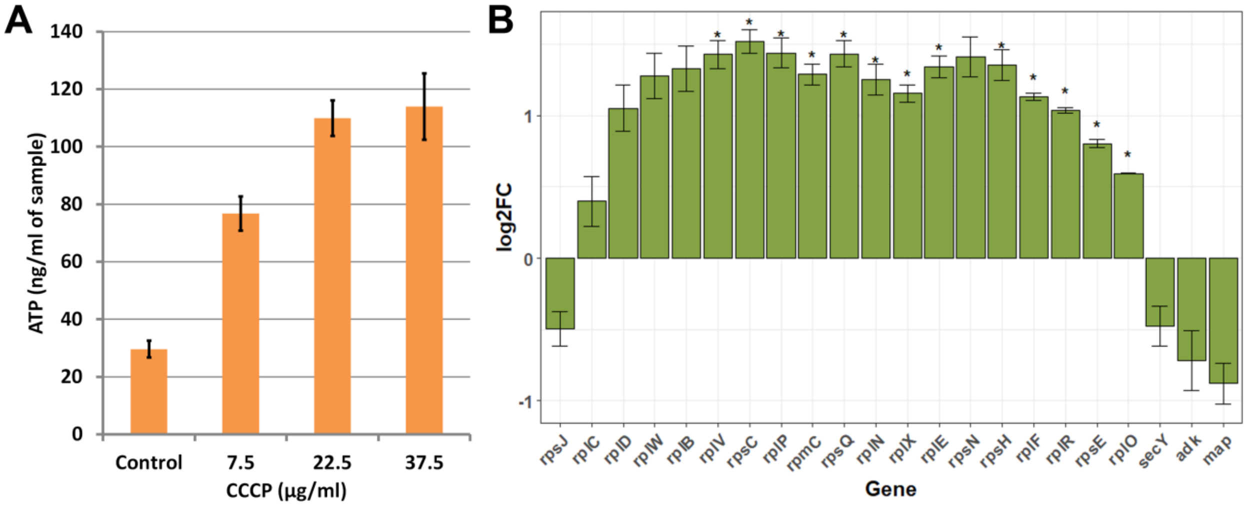

Supplementary figure S11. The effect of transient ATP concentration increase after the treatment by carbonyl cyanide 3-chlorophenylhydrazone (CCCP) on the transcription of *rpsJ* operon in *M. gallisepticum*. A – Transient increase of ATP concentration in *M. gallisepticum* under CCCP treatment. The treatment was carried out for 30 min. **B** – Differential expression of *rpsJ* operon of *M. gallisepticum* under CCCP treatment according to microarray transcriptomics data. The microarray data on *M. gallisepticum* response to CCCP treatment were obtained earlier (2). Asterisks (*) indicate differentially expressed genes with a p-value < 0.05. Standard error is indicated by error bars. Statistical analysis of microarray data was performed as described in (2).

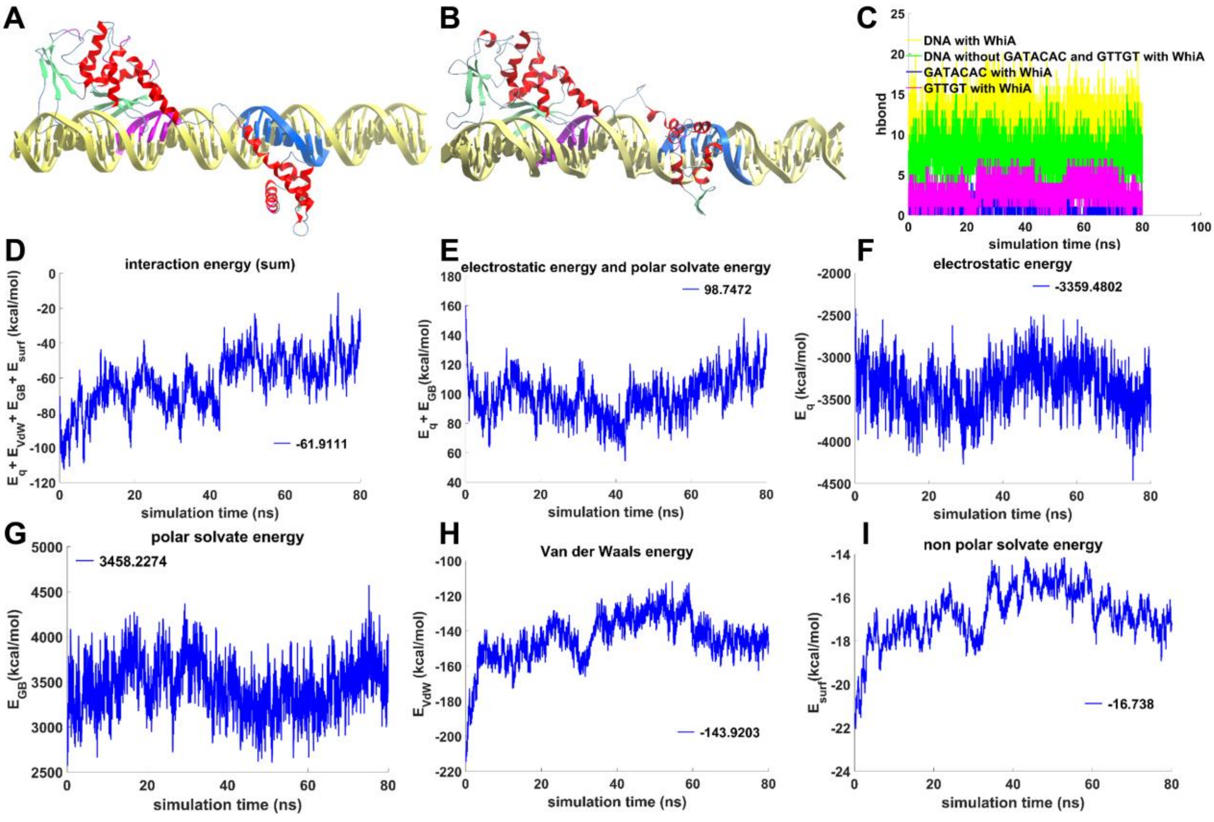

Supplementary figure S12. Molecular dynamics of WhiA binding to its recognition motif. A – Initial conformations of the molecules obtained by molecular docking prior to MD simulation. **B** – Final conformations of the molecules at the end of MD simulation. **C** – The evolution of hydrogen bonds amount during MD simulation. Colors indicate the contribution of bonds formed by WhiA with 40-mer DNA containing wild-type binding site (yellow), WhiA with DNA outside from its recognition motifs GATACAC and GTTGT (green), WhiA with core motif GATACAC (magenta), WhiA with auxiliary motif GTTGT (blue). **D** – The evolution of total interaction energy during the MD simulation. **E** – The evolution of electrostatic energy and polar solvate energy during the MD simulation. **F** – The evolution of electrostatic energy during the MD simulation. **G** – The evolution of polar solvate energy during the MD simulation. **H** – The evolution of Van der Waals energy during the MD simulation. **I** – The evolution of non-polar solvate energy during the MD simulation. (**D-I**) The calculation of binding energy was carried out using generalized Born model (GBSA). The obtained data indicate that the major contribution of the WhiA binding to DNA is paid by Van der Vaals energy and by the hydrophobic surface accessible to the solvent. Strong electrostatic interaction is leveled out by significant value of polar solvate energy. The values of parameters on the plots were smoothed using the moving average method (span = 5) and every tenth snapshot was used for these plots. The average values are indicated in the legend of each graph.

A

AA Pos	Contacts	AA	AA Pos	Contacts	AA	AA Pos	Contacts	AA	AA Pos	Contacts	AA	AA Pos	Contacts	AA
125	0	A	156	0	K	167	0	Y	210	427	D	255	4575	P
126	0	K	157	5208	L	168	7292	L	211	0	I	256	212	S
127	7217	D	158	0	I	169	0	K	212	0	V	257	7936	A
128	81	H	159	4700	T	170	8005	K->G	213	2108	N	258	5638	S->G
129	7095	H	160	227	R	171	7978	T	214	461	L	259	3221	L
130	5310	L	161	4045	K	172	7210	Q	215	0	T	260	8	A
131	0	E	162	3	N	173	67	E	216	2028	K->G	261	0	E
132	0	L	166	1	K	196	1	I	217	206	T	262	3283	L

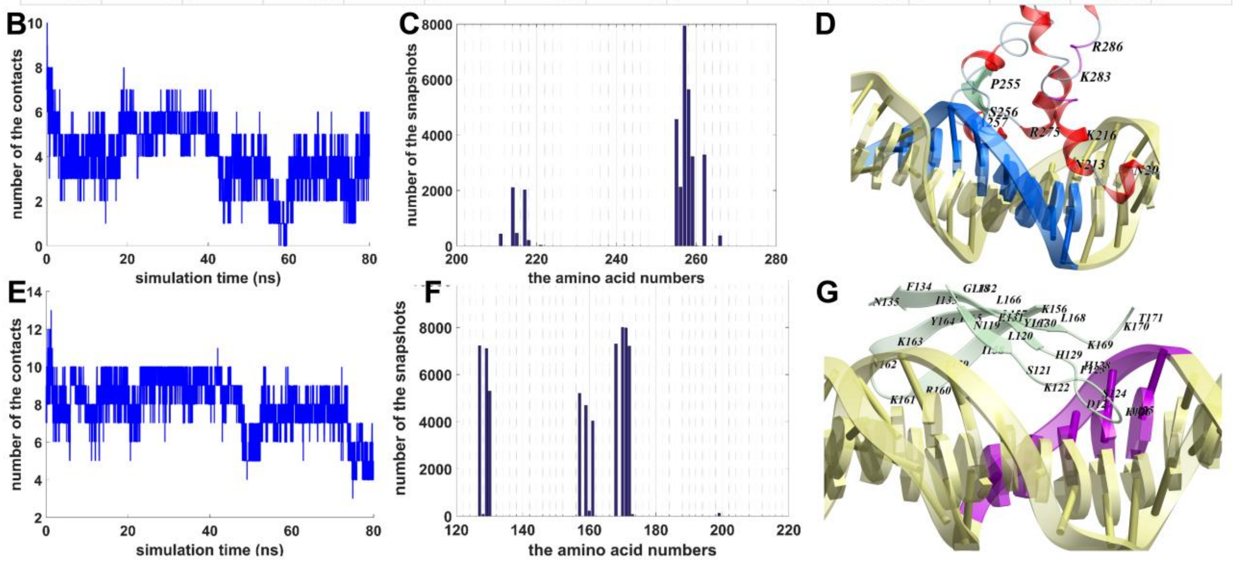

Supplementary figure S13. Identification of Mgal-WhiA aminoacid residues that pay the most contribution to the DNA binding. A – The amount of contacts between aminoacid residues with DNA. Only the regions with local maxima of the contacts are shown. They correspond to two regions within HTH domain (**C**) and three regions within HEN-domain (**F**). Blue header indicates aminoacid residues of HEN-domain; orange header indicates aminoacid residues of HTH domain. Mutation points are highlighted in red. They include Lys-170-Gly, Lys-216-Gly and Ser-258-Gly. **B** – The evolution of contacts of HTH-domain with core motif during the MD simulation. **C** – The number of snapshots during MD simulation when the aminoacid residues of HTH-domain formed contacts with core motif. The number of snapshots was used as a value characterizing the amount of contacts summarized in table (A). **D** – Snapshot of HTH-domain interaction with core motif during the MD simulation. **E** – The evolution of contacts of HEN-domain with auxiliary motif during the MD simulation. **F** – The number of snapshots during MD simulation when the aminoacid residues of HEN-domain formed contacts with auxiliary motif. The number of snapshots was used as a value characterizing the amount of contacts summarized in table (A). **G** – Snapshot of HEN-domain interaction with auxiliary motif during the MD simulation.

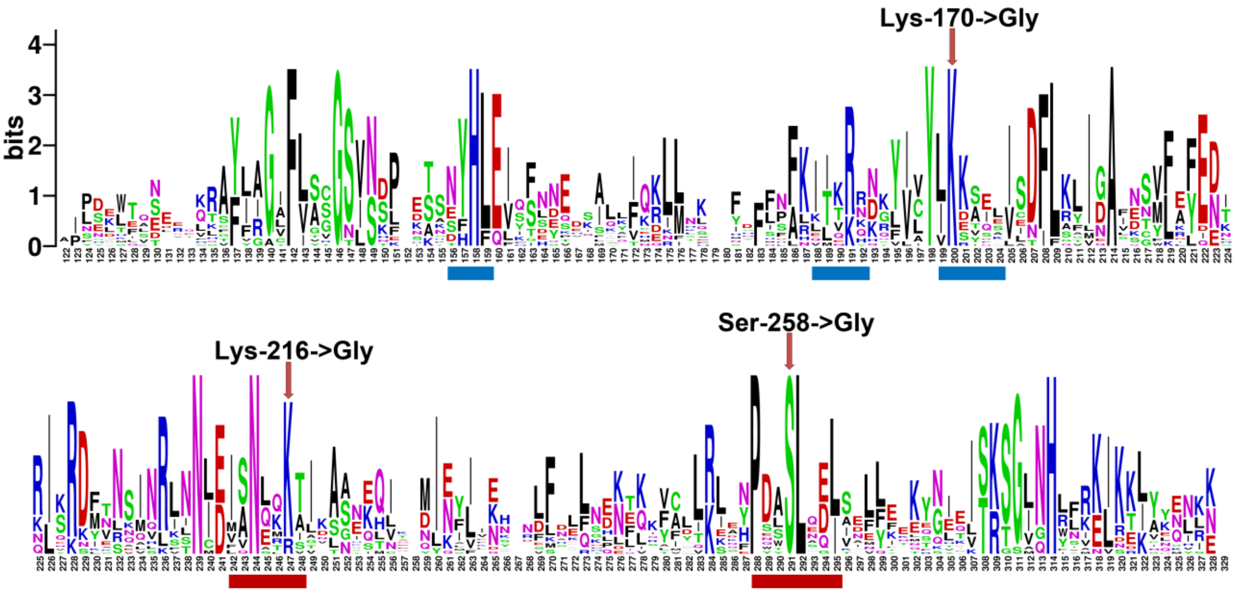

Supplementary figure S14. Conservation of WhiA sequence within the Mollicutes. The conserved regions responsible for interaction with DNA (Supplementary Fig. 4) within HEN-domain and HTH-domain are highlighted by blue (HEN) and red (HTH) colors. The highlighted regions correspond to the regions of local maxima of contacts with DNA calculated by molecular dynamics (Supplementary Fig. 13, C and F). The positions of mutation are highlighted by arrows and correspond to the most conserved aminoacid residues, e.g. the aminoacid residues that were calculated to have the local maxima of the contacts with DNA during MD simulation are the most conserved across the WhiA proteins of Mollicutes.

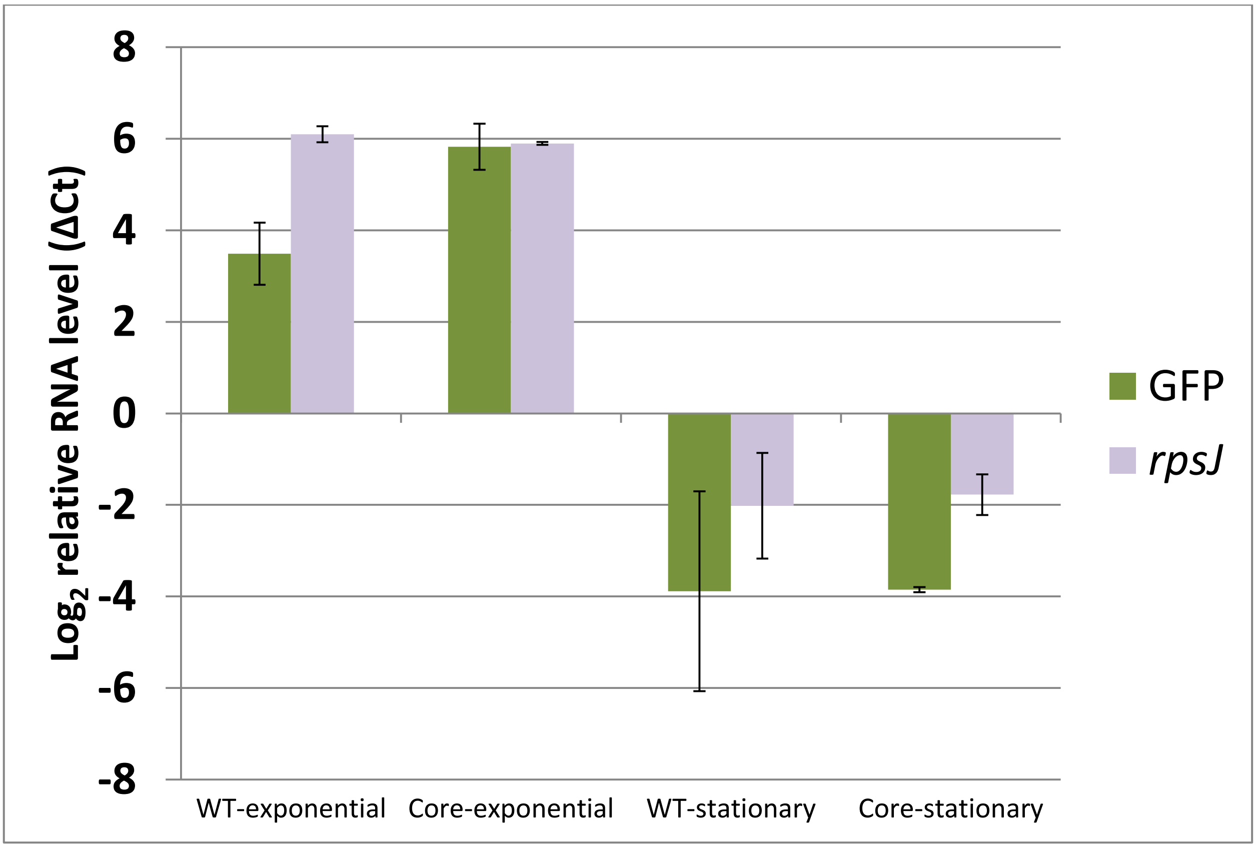

Supplementary figure S15. Transcription of *EGFP* reporter with *rpsJ* operon promoter in *M. gallisepticum*. WT – *rpsJ* operon promoter of wild-type. Core – *rpsJ* operon promoter with the mutation within the core motif, which disrupts specific WhiA binding. GFP – the RNA level of GFP gene from the reporter vector, *rpsJ* – the RNA level of genome-encoded *rpsJ* operon. Exponential – *M. gallisepticum* cells in exponential growth phase. Stationary - *M. gallisepticum* cells in stationary growth phase. The transcription of *rpsJ* operon itself is shown for comparison. RNA abundance was measured relatively to enolase transcript. Bars indicate standard error.

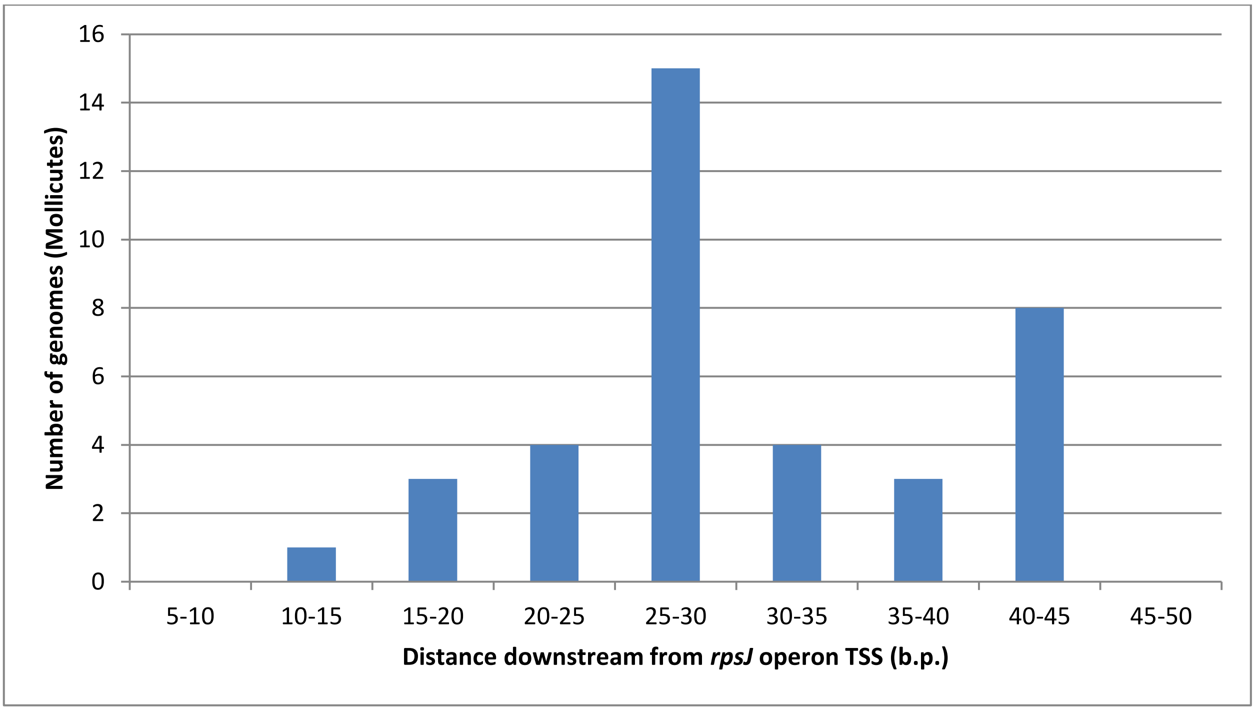

Supplementary figure S16. Distribution of distances from TSS to WhiA binding site in *rpsJ* operon promoters of Mollicutes. The TSS positions were identified from the promoter regions homology with the reference species with mapped TSSs: *M. gallisepticum*, *S. melliferum* and *A. laidlawii* (1).

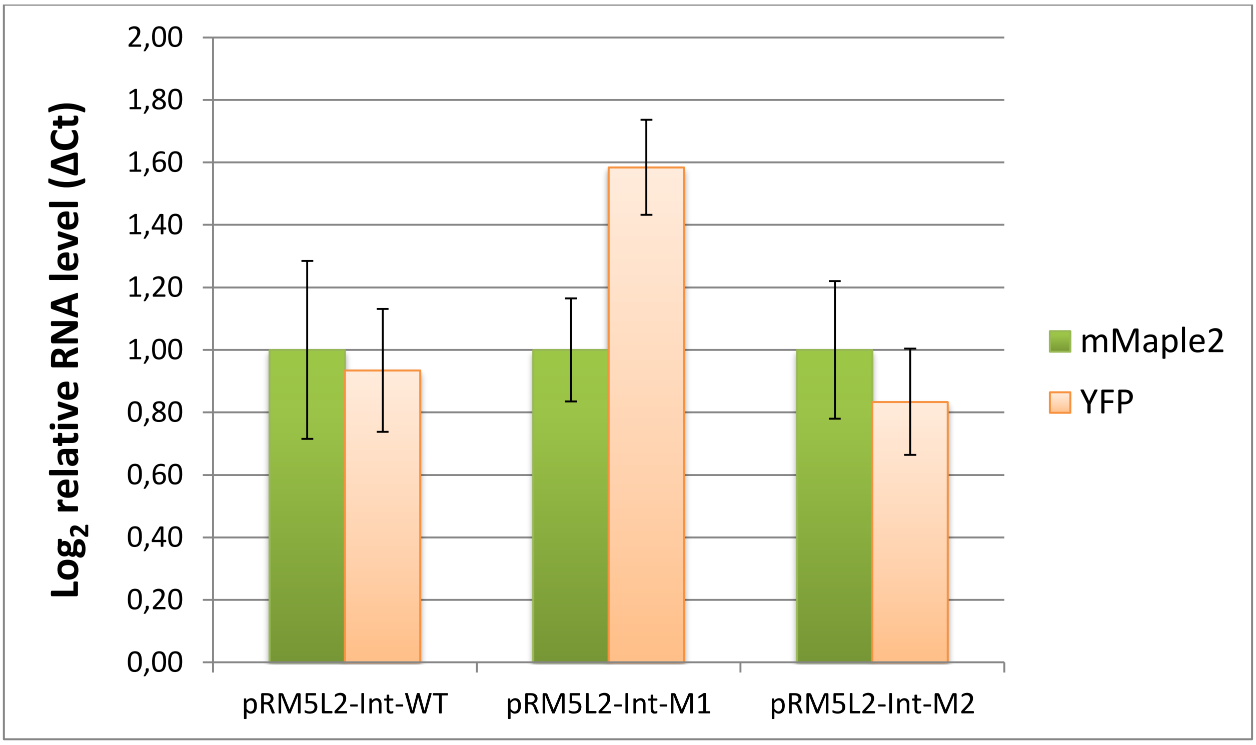

Supplementary figure S17. The effect of WhiA binding site location within operon on its transcription. An artificial operon containing mMaple2 (first) and YFP (second) coding sequences was constructed. WhiA binding site of wild-type (WT) or with mutation within the core (M1) or the auxiliary (M2) motifs was inserted between the coding sequences. The operon was transcribed from the constitutive strong promoter upstream to mMaple2 coding sequence. RNA abundance was measured relatively to enolase transcript. Bars indicate standard error.

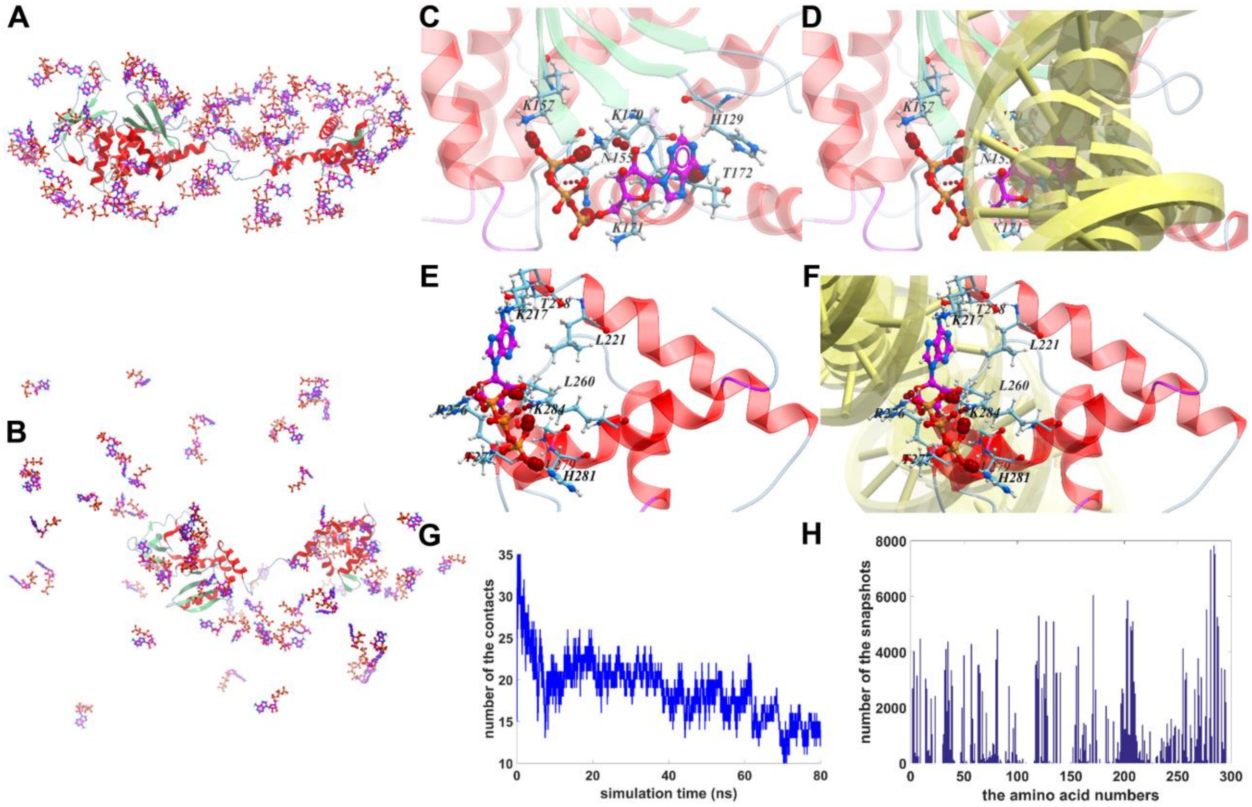

Supplementary figure S18. Molecular dynamics of ATP binding to Mgal-WhiA. Initial binding conformations of ATP molecules with Mgal-WhiA for the MD simulation (**A**) were obtained by molecular docking. The docking of ATP to Mgal-WhiA was carried out using MolSoft ICM-Pro. The procedure was carried out in two stages. First "rigid" docking was carried out. At this stage only conformational, positional and rotational flexibilities of the ligand were considered. The geometry of WhiA remained unchanged. Then "flexible" docking was carried out. At this stage the flexibilities of side chains of AA residues and of unfolded loops contacting with the ligand were considered. As a result two optimal ATP binding positions were identified: one within HEN-domain (**C, D**) and another within HTH-domain (**E, F**). Then MD simulation was performed. 70 additional ATP molecules were added into the simulation cell along with WhiA and two ATP molecules bound at the optimal sites found during the docking procedure. The additional ATP molecules were randomly distributed in the vicinity of WhiA. The same algorithm for the calculation of intermolecular contacts as for WhiA binding to DNA was used. Panels (**A)** and (**B)** show initial and final conformations of the molecules during MD simulation. The initial dense ATP surrounding of WhiA evenly distributed within the simulation cell. The amount of contacts between WhiA and ATP molecules drastically decreased during the simulation (**G**). At the same time some sites on WhiA surface retained tightly bound ATP molecules (**H**). Upon the end of MD simulation there retained two sites within WhiA with the most contacts with ATP: one within HEN-domain and another within HTH-domain. The site within HTH domain included Gly-280, Asn-283 and Lys-284. The site within HEN-domain included Lys-169, Lys-170 and Thr-172. Lys-170 was earlier identified as the residue with the local maximum of contacts with DNA. Thus it is important for both DNA recognition and ATP binding.

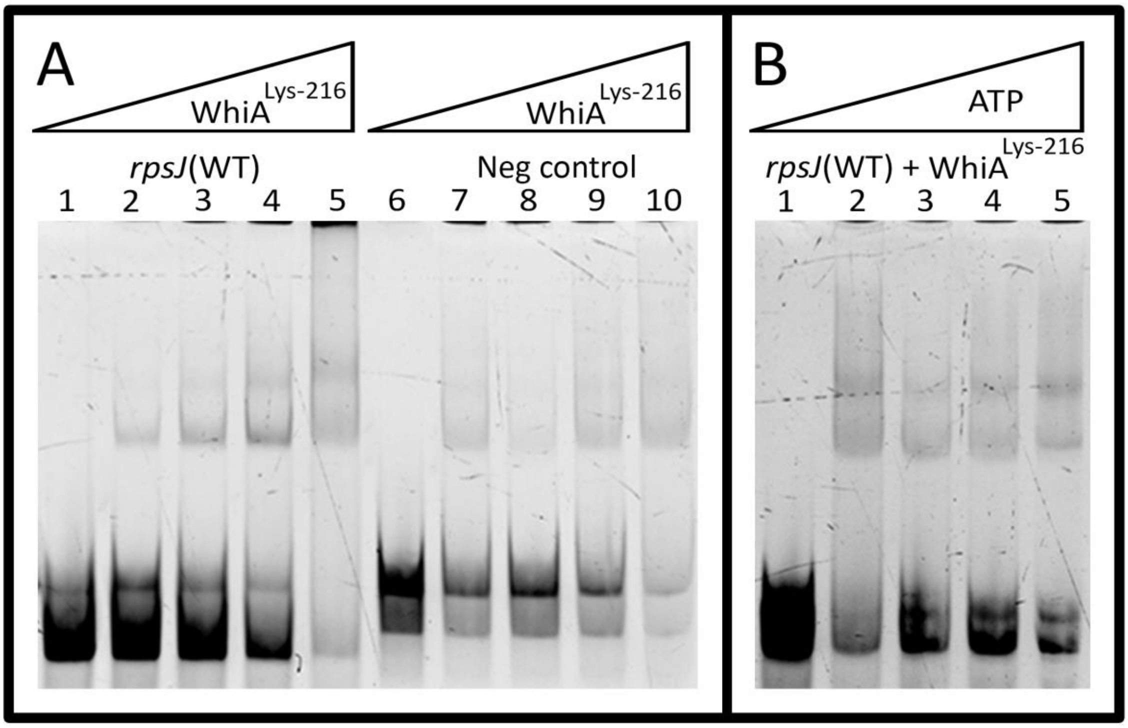

Supplementary figure S19. EMSA analysis of WhiA-Lys216 mutant. A – $WhiA^{Lys\text{-}216}$ titration, rpsJ-WT or negative control oligonucleotide 250 nM for each lane. Lanes: $WhiA^{Lys\text{-}216}$ 0 nM (negative control), 1334 nM, 2669 nM, 5338 nM, 10676 nM B – ATP titration, 5338 nM Mgal-WhiA $^{Lys\text{-}216}$ for each lane. Lanes: negative control, $WhiA^{Lys\text{-}216}$, $WhiA^{Lys\text{-}216}$+0.1 mM ATP, $WhiA^{Lys\text{-}216}$+0.5 mM ATP, $WhiA^{Lys\text{-}216}$+1 mM ATP.

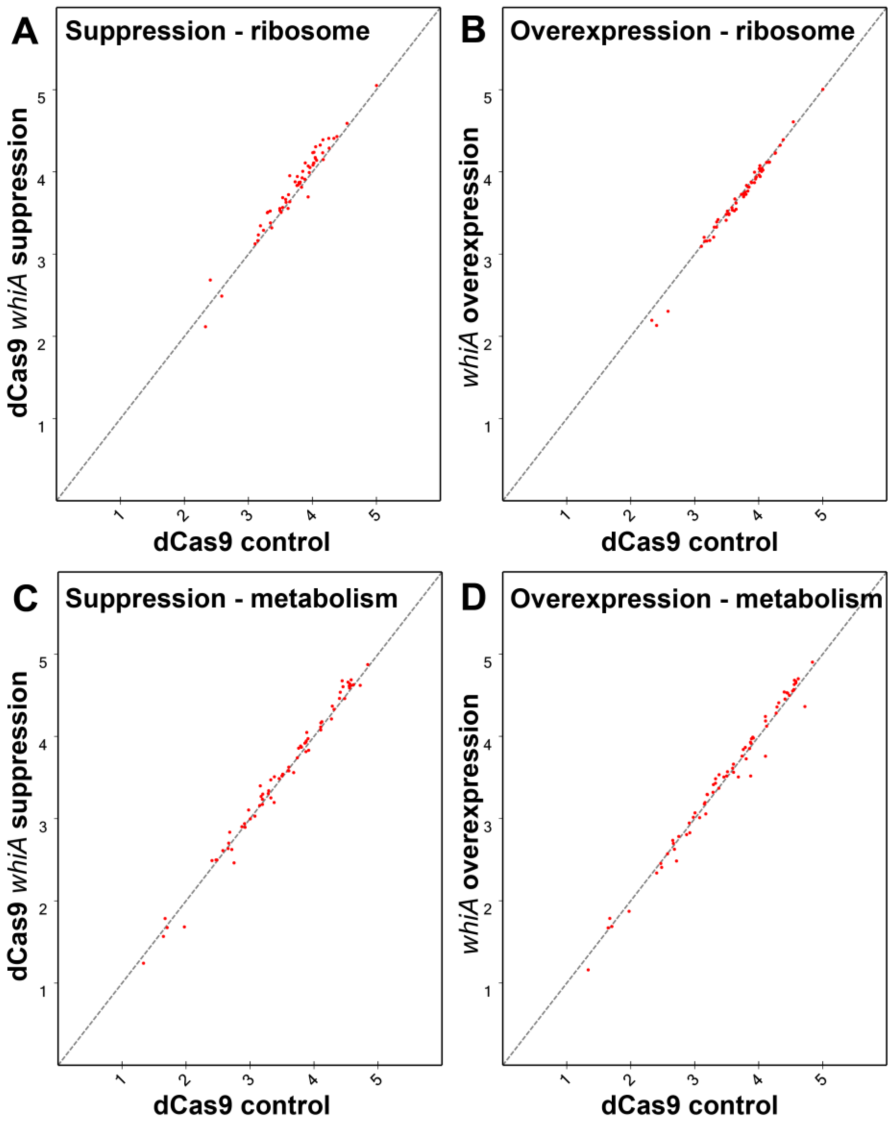

Supplementary figure S20. Systemic effect of *whiA* suppression and overexpression on *M. gallisepticum* proteome. Horizontal axis: protein abundance in dCas9 expressing strain that was used as a control. Vertical axis: protein abundance in the experimental condition, e.g. *whiA* suppression or overexpression. Protein abundances are represented as Log_2 values, average for the two strains and three biological replicates for each strain. **A** – Effect of *whiA* suppression on ribosomal constituents including ribosomal proteins and translation factors. **B** – Effect of *whiA* overexpression on ribosomal constituents. **C** – Effect of *whiA* suppression on metabolic enzymes and transporters. **D** – Effect of *whiA* overexpression on metabolic enzymes and transporters.

References

1. Fisunov GY, Garanina IA, Evsyutina D V., Semashko TA, Nikitina AS, Govorun VM. Reconstruction of Transcription Control Networks in Mollicutes by High-Throughput Identification of Promoters. Front Microbiol. 2016 Dec 6;7(1977).

2. Semashko TA, Arzamasov AA, Fisunov GY, Govorun VM. Transcription profiling data set of different states of Mycoplasma gallisepticum. Genomics Data. 2017;11:49–54.
